# Transcriptional Regulation of Sorghum Stem Composition: Key Players Identified Through Co-expression Gene Network and Comparative Genomics Analyses

**DOI:** 10.3389/fpls.2020.00224

**Published:** 2020-03-03

**Authors:** Lauriane Hennet, Angélique Berger, Noemi Trabanco, Emeline Ricciuti, Jean-François Dufayard, Stéphanie Bocs, Denis Bastianelli, Laurent Bonnal, Sandrine Roques, Laura Rossini, Delphine Luquet, Nancy Terrier, David Pot

**Affiliations:** ^1^CIRAD, UMR AGAP, Montpellier, France; ^2^CIRAD, INRA, Montpellier SupAgro, University of Montpellier, Montpellier, France; ^3^Parco Tecnologico Padano, Lodi, Italy; ^4^Centro de Biotecnología y Genómica de Plantas, UPM-INIA, Instituto Nacional de Investigación y Tecnología Agraria y Alimentaria, Madrid, Spain; ^5^CIRAD, UMR SELMET, Montpellier, France; ^6^Department of Agricultural and Environmental Sciences - Production, Landscape, Agroenergy, Università degli Studi di Milano, Milan, Italy; ^7^AGAP, CIRAD, INRAE, Montpellier SupAgro, University of Montpellier, Montpellier, France

**Keywords:** cell wall, gene co-expression network analysis, internode, phylogeny, transcription factor, NAC, MYB, sorghum

## Abstract

Most sorghum biomass accumulates in stem secondary cell walls (SCW). As sorghum stems are used as raw materials for various purposes such as feed, energy and fiber reinforced polymers, identifying the genes responsible for SCW establishment is highly important. Taking advantage of studies performed in model species, most of the structural genes contributing at the molecular level to the SCW biosynthesis in sorghum have been proposed while their regulatory factors have mostly not been determined. Validation of the role of several MYB and NAC transcription factors in SCW regulation in Arabidopsis and a few other species has been provided. In this study, we contributed to the recent efforts made in grasses to uncover the mechanisms underlying SCW establishment. We reported updated phylogenies of NAC and MYB in 9 different species and exploited findings from other species to highlight candidate regulators of SCW in sorghum. We acquired expression data during sorghum internode development and used co-expression analyses to determine groups of co-expressed genes that are likely to be involved in SCW establishment. We were able to identify two groups of co-expressed genes presenting multiple evidences of involvement in SCW building. Gene enrichment analysis of MYB and NAC genes provided evidence that while NAC SECONDARY WALL THICKENING PROMOTING FACTOR NST genes and SECONDARY WALL-ASSOCIATED NAC DOMAIN PROTEIN gene functions appear to be conserved in sorghum, NAC master regulators of SCW in sorghum may not be as tissue compartmentalized as in Arabidopsis. We showed that for every homolog of the key SCW MYB in Arabidopsis, a similar role is expected for sorghum. In addition, we unveiled sorghum MYB and NAC that have not been identified to date as being involved in cell wall regulation. Although specific validation of the MYB and NAC genes uncovered in this study is needed, we provide a network of sorghum genes involved in SCW both at the structural and regulatory levels.

## Introduction

Sorghum is the fifth ranking cereal crop in the world in terms of grain production behind wheat, barley, maize and rice. At the global level, almost half a billion people rely on sorghum daily and consume sorghum grain and flour as a staple food (Paterson, 2008; Frère et al., 2011). In addition, sorghum grains and vegetative parts are also intensively used for feed, energy and natural fiber-reinforced polymer production (Antonopoulou et al., 2008; Carpita and McCann, 2008; Dien et al., 2009; Mullet et al., 2014; Anami et al., 2015; Barcelos et al., 2016; Brenton et al., 2016; Carvalho and Rooney, 2017; Thomas et al., 2019).

Sorghum is a C4 crop that is able to efficiently fix carbon and produce large amounts of vegetative biomass in many different agrosystems. Due to the tolerance of sorghum to low inputs, temperature variability and drought stress (Sanderson et al., 1992; Rooney et al., 2007; Schmidhuber and Tubiello, 2007; Brown et al., 2008; Zegada-Lizarazu et al., 2012), this crop emerges as a relevant candidate to provide the raw material required for energy and bio based material production. Furthermore, sorghum presents several assets as a plant model (Swaminathan et al., 2010; Aitken et al., 2014; Mullet et al., 2014). The genome of sorghum is relatively simple compared to other grasses with a 730 Mb size unaffected by any recent genome duplication (Paterson et al., 2009; Schnable et al., 2011). Sorghum also exhibits a large genetic (Billot et al., 2013; Morris et al., 2013; Sekhon et al., 2016) and phenotypic diversity, which are extensively mobilized in temperate and tropical breeding programs. Finally, transformation and genome editing tools are available (Liu and Godwin, 2012; Wu et al., 2014; Che et al., 2018).

In sorghum, most of the shoot biomass is allocated to the stem in the form of soluble sugars and cell walls (CW). In this article, we focus on the secondary cell wall (SCW) which accounts for a great share of this biomass and is the main resource for energy, natural fiber-reinforced polymer production and animal nutrition. Sorghum SCW is composed of approximately 50% of cellulose, 43% of hemicelluloses, these polysaccharides being also present in the primary cell wall (PCW), and 7% of lignins, a polymeric phenolic compound specific to SCW (Trouche et al., 2014). These CW elements accumulate around fully grown cells that form conductive vessels but also around sclerenchymatous cells, which are the fibers supporting the stem. Many traits of adaptive and agronomic interest rely on the composition of the SCW deposited around stem cells such as plant standability (Gomez et al., 2017, 2018), water transport and biotic and abiotic stress resistance. In addition to these general concerns, as the stem is the raw material for several different uses, SCW composition plays a key role in the adaptation of the varieties to different end-uses. However, breeders struggle to develop novel varieties gathering desired and sometimes discordant target traits such as plant stiffness, polysaccharide content or stem digestibility. Understanding the mechanisms of SCW formation may unable the enhancement of selection efficiency to meet the needs of growers and users.

In sorghum, significant advances have been achieved through analyses of natural and induced mutants. Genes inducing the brown midrib (*bmr*) phenotype, which is accompanied by a higher degradability of the CWs have primarily been investigated. To date, a total of 8 *bmr* genes have been discovered in sorghum (Saballos et al., 2012; Sattler et al., 2014). Three of these genes have been characterized at the molecular level and correspond to enzymes of the lignin biosynthetic pathway. Some of these genes have been extensively used to develop sorghum varieties targeting the feed industry (Pedersen et al., 2008). In addition to the *bmr* phenotype, additional phenotypic mutants related to the composition of the SCW have been identified. Petti et al. (2013, 2015) identified that *REDforGREEN* and *dwarf1.1* mutants affected lignin and cellulose abundance in leaves and stems, in addition to their respective red coloration and shortened internodes.

Although biparental (Murray et al., 2008a, b; Shiringani and Friedt, 2011) and broad-based population analyses (Brenton et al., 2016; Li et al., 2018) allowed the identification of candidate genomic regions potentially contributing to the variability of SCW components, these approaches failed to provide an exhaustive understanding of the genetic control of SCW composition variability in sorghum. At the same time, several transcriptomic analyses attempting to elucidate the molecular pathways and mechanisms underlying SCW establishment in developing internodes highlighted the differential expression patterns of extensive gene sets (Shakoor et al., 2014; McKinley et al., 2016; Rai et al., 2016; Kebrom et al., 2017). Nevertheless low levels of congruence between the genetic (Quantitative Trait Loci/Quantitative Trait Nucleotides) and genomic (transcriptomic) approaches have been observed to date. Only a few structural candidate genes controlling the activity of key CW biosynthetic enzymes were identified and additional work is needed regarding the transcription factors (TF) that can fine-tune the mechanisms involved in SCW deposition.

**FIGURE 1:**
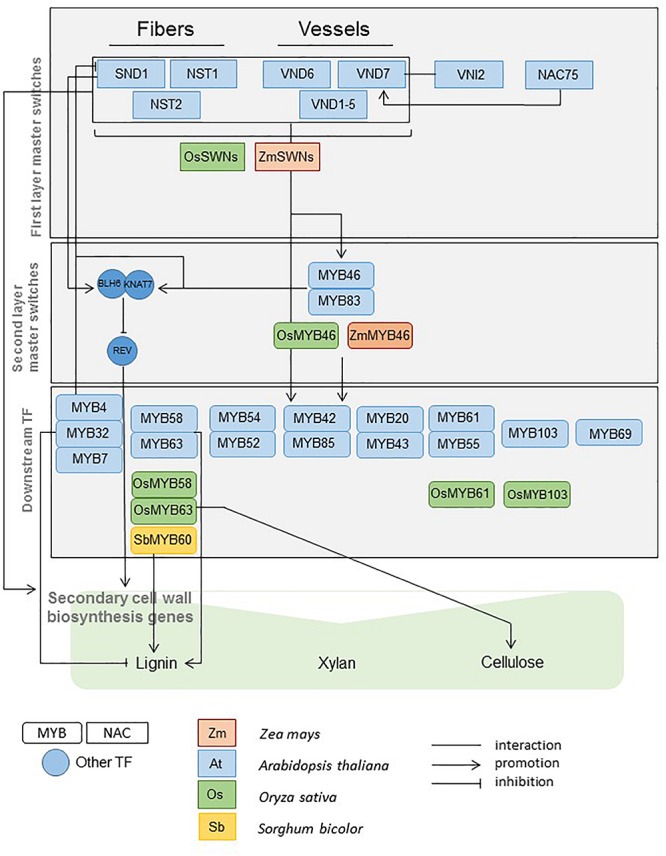
MYB, NAC, and other regulators of SCW biosynthesis functionally validated in Arabidopsis, maize, rice, and sorghum (Adapted from Wang and Dixon, 2012; Liu et al., 2014; Rao and Dixon, 2018).

Previous work in Arabidopsis provided strong knowledge on the TFs involved in SCW regulation. Most of these genes are members of the MYB (Myb proto-oncogene like) and the NAC (NAM, ATAF and CUC2) TF families (Zhong et al., 2008). These TFs act as master switches of SCW deposition or as more specific regulators of SCW component synthesis and assembly (Figure 1). The functions of some of the master regulators have been shown to be conserved, at least in some aspects, in rice, maize, poplar and eucalyptus (Goicoechea et al., 2005; McCarthy et al., 2010; Zhong et al., 2011a, b, 2013). The TF knowledge base that has been developed in Arabidopsis and in a few other model species constitutes an opportunity to accelerate and facilitate the discovery of genes involved in SCW regulation in sorghum and grasses. Regulation of SCW deposition in sorghum is only scarcely understood. Only one TF (SbMyb60) has been recently validated to induce monolignol biosynthetic pathway (Scully et al., 2016) (Figure 1).

The objective of this work is to identify regulatory mechanisms of SCW deposition in sorghum, First, the homologies between TF already validated for their role in SCW deposition regulation in various species (grass and non-grass) and sorghum genes have been investigated using comparative genomics. In a second step, transcriptomic datasets based on internode development dynamics were produced and used to reach a better understanding of the co-expressed gene networks and key TF s likely to be involved in SCW formation.

## Materials and Methods

### Phylogenetic Analyses of the MYB and NAC Transcription Factor Families

Publicly available MYB and NAC protein sequences^1^ from *Arabidopsis thaliana* were used to produce specific signatures of each family using the Galaxy toolbox (Afgan et al., 2018) with MAAFT (Katoh et al., 2017) and TrimAl scripts (Capella-Gutiérrez et al., 2009) (only sites with less than 90% of gaps were kept, conserving at least 10% of the total protein length, otherwise the default parameters were used). As a second step, highly specific thresholds for protein sequence similarity (*e*-value = 10^–22^ for the MYB family and *e*-value = 10^–30^ for NAC family, these thresholds were deduced from observed *e*-values of Arabidopsis) were used to recover homolog proteins from 9 plant proteomes using the Arabidopsis signatures library and the HMM (Hidden Markov Model) approach. Five monocotyledons (maize: *Zea mays*, rice: *Oryza sativa susp japonica*, sorghum*: Sorghum bicolor*, purple false brome: *Brachypodium distachyon*, and foxtail millet: *Setaria italica*) and four dicotyledon proteomes (*Arabidopsis thaliana*, barrel medic*: Medicago truncatula*, eucalyptus*: Eucalyptus grandis*, and poplar: *Populus trichocarpa*) were used in this step.

Then, alignments of full-length proteins were generated with MAFFT and the sequences were trimmed using Trimal (gt option = 0.9 and cons option = 10). The alignments were refined keeping only the longest splicing variant of each gene. One phylogenetic tree per TF family was generated using the Maximum Likelihood method implemented in PhyML (Guindon et al., 2010; Anisimova et al., 2011; Zhou et al., 2018). The Rap-Green rooting tool (Dufayard et al., 2005) was used to reconcile the obtained gene trees with the general species tree to identify gene duplication and optimize gene function inference. Full size phylogenetic trees are available in the Supplementary Material as well as online^2^.

### Gene Expression Evolution During Internode Development of a Sorghum Biomass Hybrid in Different Water Availability Regimes

#### Field Conditions and Sampling Strategies

Transcriptome evolution of developing internodes was analyzed in three field experimental trials in 2013, 2014, and 2015. In these three experiments, “Biomass140,” a commercial hybrid developed by Eurosorgho^3^^4^, which corresponds to an industrial biomass ideotype (high biomass production and late flowering), was analyzed in contrasting water availability regimes on the DIAPHEN field phenotyping platform in Mauguio (southern France; Delalande et al., 2015; 43°36′43′′N, 3°58′2′′ E) during the summer seasons (sowing on May 22nd, May 23rd, May 13th, respectively for 2013, 2014, and 2015).

The water regimes corresponded to the well-watered (WW) and water deficit (WD) treatments. WD consisted of a 1-month dry-down period that began when the plants had, on average, 11 ligulated (expanded) leaves on the main stem. The stage of 11 ligulated leaves was chosen because it corresponds to the onset of rapid elongation of internodes (Gutjahr et al., 2013).

Although the main objective of this work was to reach a better understanding of the molecular mechanisms underlying cell wall establishment in sorghum, we decided to take advantage of the results produced in two contrasting water regimes. This decision was motivated by the facts that gene co-expression networks relevance relies on the number of biological conditions that are explored (taking advantage of the WD samples we doubled the biological conditions explored), and on the comparability of the samples analyzed (in our case, we focused our attention on internode samples exclusively). As the objective of this study was not to identify the genes involved in the specific response to WD but instead to highlight the gene co-expression networks constitutively involved in the establishment of the internode cell wall, neither differential expression analysis nor identification of gene networks activated in response to drought deficit were performed.

The experiments corresponded to randomized complete block designs with 4 and 3 replications in 2013 and 2014–2015, respectively. The individual plot had 7 m long rows spaced at 0.8 m (8 and 4 rows per plot respectively in 2013 and 2014–2015). Eighteen seeds were sown per linear meter.

Different sampling strategies of the internodes were used for the three experiments (Supplementary Figure S1). In 2013, two internode levels corresponding to the 12^th^ and 16^th^ ligulated leaves were harvested at five different stages (defined as D1 to D5) in the two water regimes (with the exception of the top internode for the first stage as it was not yet available). In 2014, six internode levels were harvested at three different stages in the two water regimes. The internodes corresponding to the 13^th^ and 15^th^ ligulated leaves were harvested at the three sampling stages. The internodes corresponding to the 14^th^ and 16^th^ ligulated leaves were harvested only for the first sampling stage and the internodes corresponding to the 17^th^ and 19^th^ ligulated leaves were exclusively harvested at the last two sampling stages. In 2015, four internode levels were harvested at two stages in the two water regimes. In this case, the internodes were sampled according to their relative position from the last ligulated leaf of the stem. For the first date of analysis (23/07/2015), the four top internodes starting from the last ligulated leaf were harvested. On average, the internodes corresponded to the internodes ranked 14^th^, 13^th^, 12^th^, and 11^th^. For the second date of analysis (24/09/2015), the four internodes that were sampled corresponded to the flag leaf internode (FL, which corresponded on average to the 23^rd^ internode rank) to the FL-2 (21^st^ internode rank), FL-4 (19^th^ internode rank) and FL-6 internodes (17^th^ internode rank). Each internode sample corresponded to pools of 3 or 4 individual internodes from independent plants, respectively for 2014–2015 and 2013. Duplicated internode samples were harvested for each internode level, stage, biological replicate and either directly frozen in liquid nitrogen and stored at −80°C after harvest for further transcriptomic analysis or dried at 60°C for 72 h for biochemical composition analyses.

#### Total RNA Isolation and Library Construction

Total RNA isolation was performed according to the procedure described in Chomczynski and Sacchi (1987). Frozen internodes (−80°C) were ground to a fine powder using the IKA^®^ A11 basic analysis mill (Ika, Staufen, Germany) and 1 ml of TRIZOL^®^ Reagent was used for 100 mg powder. RNA integrity number (RIN) and quantification of total RNA were measured using the Agilent 2100 Bioanalyzer Nano 6000 chip. A total of 398 RNASeq libraries (158 in 2013, 144 in 2014 and 96 in 2015), for a total of 191 internode samples, were prepared according to the Illumina protocol with the TruSeq RNA Library Prep Kit (Illumina, United States), using 1 μg of total RNA. The indexed libraries were pooled in 24-plex and subjected to single-end 1 × 150 bp sequencing on an Illumina HiSeq2500 (at the Genotoul platform^5^ in Toulouse, France). Each pool of 24 libraries was run in parallel on two lanes, allowing a technical replicate (a total of 14 lanes). The raw sequence files are available on the short read archive under the bioproject PRJNA560153.

#### Quality Control, Alignment and Read Count Definition

FastQC (Andrews, 2010) was used to check raw read sequence quality [i.e., detection of adaptors, average quality on the whole sequence (phred score), GC content, duplicated reads and PCR contamination]. Fastq reads were cleaned using cutAdapt (Martin, 2011) to trim read ends of poor quality (*Q* score below 20) and to keep only those with an average quality above 30 and a minimum length of 35 base pairs. For the following steps, the programs described in Sarah et al. (2017) and available on the Southgreen platform^6^ were used: arcad_hts_0_fastqc_in_chains.pl, arcad_hts_1_cutadapt_in_chain.pl, and arcad_hts_2_Filter_ Fastq_On_Mean_Quality.pl.

Transcript expression levels have been estimated with the new Tuxedo pipeline (Pertea et al., 2016). First, for each RNA sample, RNA-seq reads were mapped on the sorghum genome assembly Sbicolor_313_v3.1 using Hisat2 (Kim et al., 2015). Genes and transcripts were assembled and quantified with stringtie, using the reference annotation file (in GFF3 format) to guide the assembly process. The output included expressed reference transcripts as well as any novel transcripts that were assembled. Gffcompare^7^ was used to compare transcripts with the reference annotation (gene and transcript predictions) and to identify new genes/transcripts. The sorghum genome assembly “Sbicolor_313_v3.1” and enriched annotation files have been used to estimate abundance with stringtie.

### Gene Co-expression Network Analysis

#### Identification of the Co-expression Gene Networks

Only genes harboring more than 3 reads in at least 20 internode samples (over the 191 internode samples) were considered for the co-expression analyses. The co-expression gene network was built with the WGCNA R package (Langfelder and Horvath, 2008, 2012) using the normalized and filtered expression data set. Normalization was performed with the EdgeR package. A total of 20.294 genes were finally retained in the co-expression network analysis. Block and year effects were estimated using the removeBatchEffect function from the R Limma package (Ritchie et al., 2015). No such effect was detected and no outlier samples needed to be trimmed. The network was built using the “signed” networkType parameter, enabling to capture the direction of the expression variation and grouping genes with the same direction variation in gene expression. This parameter is advised to identify biologically meaningful modules (van Dam et al., 2018). According to the mean connectivity and the scale-free topology index curves (Supplementary Figure S2), a power of 12, which is commonly used for this type of data, was used in this study. This result showed that our data fit the scale-free topology approximation, which is desirable to use WGCNA analysis. It also indicated that no strong driver could bias our analysis. Relationships between every pair of genes were explored with Pearson correlation coefficient in the WGCNA package. For most of the results discussed, only genes harboring strong correlations with other genes were considered. An adjacency (which is the correlation raised to a power allowing to amplify disparity between strong and weak correlation) threshold of 0.1, corresponding to an expression correlation of 0.82 between genes was used to define the different modules. Otherwise default parameters of WGCNA were used and modules were named with randomly picked colors.

#### Gene Network Enrichment Analysis

As a global approach, the modules obtained using WGCNA were characterized according to their enrichment in Gene Ontology (GO) terms using the TopGO R package (Alexa and Rahnenfuhrer, 2019). Default parameters and the sorghum GO list from PLAZA monocots 4.0 database^8^ were used. A threshold p-value of 0.05 was considered to define significant enriched ontologies. Raw enrichment results are available in Supplementary Table S1.

We gathered Biological Process GO terms in keyword groups to address easily meaningful information (Supplementary Table S2). For instance, under the keyword “SCW” we gathered four different ontologies; negative/positive “regulation of SCW biogenesis,” “regulation of SCW biogenesis,” and “plant-type secondary cell biogenesis.”

A custom gene list of CW related genes was obtained from the aggregation of genes listed in previous publications (Petti et al., 2015; McKinley et al., 2016; Rai et al., 2016) (Supplementary Table S3). In addition to the GO enrichment analysis, Fisher’s exact test was also performed for every module to detect enrichment in these candidate CW related genes.

In addition, the sorghum MYB and NAC genes (SbMYB and SbNAC) identified in phylogenetic analyses were used as bait genes to characterize their co-expressed gene networks. For this purpose, the 15 most correlated genes were selected for every MYB or NAC gene. Then, a second layer of co-expressed genes was retrieved by listing the top 15 correlated genes of the first layer. These so called “top subnetwork gene lists” were analyzed using the GO term and CW related gene enrichment analyses presented earlier. Lists of genes for each bait gene and their significant enrichment are available as Supplementary Data (Supplementary Tables S4, S5).

#### Identification of Hub Genes

Intramodular connectivity of each gene was calculated using the WGCNA function “intramodularConnectivty.fromExpr”. The highest connectivity is, the more central the gene is. These top genes are expected to play crucial biological roles. We compared the “top 10%” and top 10 genes in the whole considered module (i.e., without correlation threshold selection) with the “top 10%” and top10 genes preliminarily filtered considering the 0.82 correlation threshold.

#### Internode Biochemical Characterization

The quantification of lignin, cellulose and hemicellulose contents were derived from Near InfraRed (NIR) Spectrum analysis based on the Van Soest reference method (Van Soest et al., 2010). This method provides estimates of total fiber (NDF, neutral detergent fiber, expressed in percentage of dry matter, %DM), lignocellulose (ADF, acid detergent fiber, expressed in %DM) and lignin (ADL, acid detergent lignin, expressed in %DM). The same internode levels as those used for the transcriptomic analyses were sampled and dried for 72 h at 60°C. The dried samples were ground at a 1 mm sieving size and NIR spectra were acquired with a NIR system 6500 spectrometer (FOSS NirSystem, Laurel, MD, United States). The calibration models for the different traits were developed according to a set of more than 700 reference biochemical data points for each trait. NDF, ADF, and ADL were then used to calculate the hemicellulose (Xylans) content (computed as NDF-ADF, %DM) and the cellulose content (computed as ADF-ADL, %DM). The Crop Ontology ID^9^ of the variables used in this study are provided in Supplementary Table S6.

## Results

### Phylogenetic Analyses of the NAC Transcription Factor Family

NAC genes from five monocotyledons (sorghum, maize, rice, foxtail millet, purple false brome) and four dicotyledons (Arabidopsis, poplar, eucalyptus, barrel medic) were retrieved from the nucleotide databases. One hundred twelve Arabidopsis genes, 168 poplar, 163 eucalyptus, 70 barrel medic, 136 maize, 132 foxtail millet, 129 rice, 138 brome and 122 sorghum genes were identified (Supplementary Table S7). These numbers are relatively similar to those previously reported in these species. Previously, 105 (Ooka et al., 2003) and 117 (Nuruzzaman et al., 2010) NAC genes were identified in Arabidopsis, 288 (Pereira-Santana et al., 2015) and 163 (Hu et al., 2010) in poplar, 189 in eucalyptus (Hussey et al., 2015), 97 in barrel medic (Ling et al., 2017), 124 (Fan et al., 2014) and 152 (Shiriga et al., 2014) in maize, 147 in foxtail millet (Puranik et al., 2013), 151 (Nuruzzaman et al., 2010) and 140 (Fang et al., 2008) in rice, 107 (You et al., 2015) and 118 (Zhu et al., 2015) in brome and 131 (Sanjari et al., 2019) and 145 (Kadier et al., 2017) in sorghum.

**FIGURE 2:**
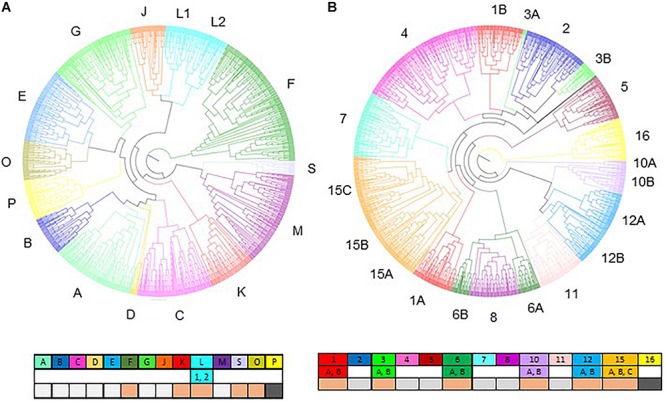
Phylogenetic trees of NAC **(A)** and MYB **(B)** based on the protein sequences from five monocotyledons and four dicotyledons species. Colors correspond to the different clades (letters for NAC and numbers for MYB, see caption). Subclades are designed with numbers in the NAC tree and letters in the MYB tree. Comparisons with the phylogenetic trees obtained by Sanjari et al. (2019) for the NAC tree and with Li et al. (2016) for the MYB tree are provided below the clade identifiers. Conserved clades are indicated by light gray boxes, new clades are in dark gray and clades with reallocation of genes are in light orange.

We used a recent study of the NAC family in sorghum (Sanjari et al., 2019) to support the characterization of the phylogenetic tree obtained in this work. We recovered 122 out of 131 SbNAC reported in Sanjari’s work and eight of the 15 clades described by Sanjari et al. (2019) remained unchanged in our tree (Figure 2A, Supplementary Table S7, and Supplementary Figure S3). The former clade N was fused to the former clade O becoming the new clade O. Clade K was divided into new clades S and K. Clade L was divided into two subclades 1 and 2. New clade F gathered most of the former clade F genes, some genes from different former clades and all genes from former clades I and clade H that no longer exist. Among the nine genes reported by Sanjari et al. (2019) that were not identified in the present analysis, seven were former clade F genes. Outside of clade F genes, only four sorghum genes did not match the former clade and were allocated to new clades. A new clade P was created and contains only monocotyledon genes. Enrichments of clades B and F in monocotyledon genes were observed. Clade F which is the largest one detected, with 193 sequences, contains 135 (69%) monocotyledon and 58 (31%) dicotyledon genes. No functional information is available in Arabidopsis regarding this clade. Among the 54 genes included in clade B, 81% (44 genes) correspond to monocotyledon genes. According to the function reported for AtNAC1 which belongs to this clade, clade B can be potentially involved in auxin transportation (Xie et al., 2000).

Among the thirteen Arabidopsis NAC genes for which functional evidence of involvement in CW establishment has been reported, nine belong to the C clade, whereas two are included in the G clade (SECONDARY WALL-ASSOCIATED NAC DOMAIN PROTEIN 2 AtSND2 and AtSND3) and one is included in clade K (VND-INTERACTING 2 AtVNI2).

**FIGURE 3:**
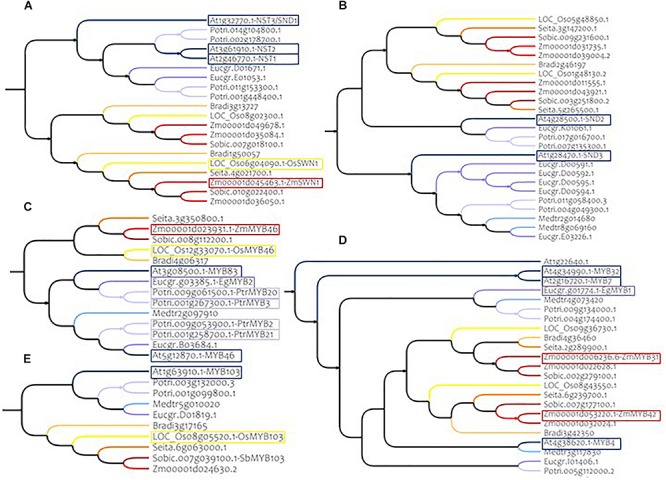
Phylogenetic subtrees of some major MYB and NAC genes in nine species. **(A)** Homologs of AtNST1, AtNST2, and AtNST3/SND1 (Clade C) **(B)** homologs of AtSND2 and AtSND3 (Clade G) **(C)** homologs of AtMYB83 and AtMYB46 (Clade 4) **(D)** homologs of AtMYB4 (Clade 15A) **(E)** homologs of AtMYB103 (Clade 4). Warm colors (yellow to red) represent monocotyledon branches (rice, maize, setaria, brachypodium, and sorghum) and cold colors (gray to dark blue) represent dicotyledon branches (medicago, eucalyptus, poplar and Arabidopsis). Colored frames highlight functionally validated genes. Correspondences between sorghum genes IDs and gene name synonyms are available in Supplementary Table S11.

Overall, according to the specific monocotyledon and dicotyledon lineages detected for the different SCW NAC genes, no single specific sorghum ortholog of the AtNAC genes was identified. With the exception of VNI2 (At5g13180) for which a large diversification was observed in monocotyledons (6 homologs in sorghum and up to 13 in Brachypodium, Supplementary Figure S4A), a relative homogeneity of the gene numbers was observed between Arabidopsis and sorghum. As an example, we identified two sorghum orthologs (here referred as SbNSTa and SbNSTb, Figure 3A and Table 1) for the Arabidopsis genes AtNST1, AtNST2 and AtNST3/SND1 (NAC SECONDARY WALL THICKENING PROMOTING FACTOR1, 2 and 3). These closely related genes are responsible for SCW regulation in Arabidopsis (Mitsuda et al., 2005, 2007; Zhong et al., 2006). We also identified two sorghum orthologs for genes AtSND2 and AtSND3 (here referred as SbSNDa and SbSNDb, Figure 3B and Table 1), that have been described as SCW regulators in Arabidopsis too (Zhong et al., 2008; Hussey et al., 2011; Sakamoto and Mitsuda, 2015).

**TABLE 1:** Major MYB and NAC regulators of SCW in Arabidopsis, their homolog genes in sorghum and their homolog genes validated in other species.

**TF**	**Arabidopsis SCW gene**				**Homologs**			
	**Gene ID**	**Name synonym**	**Publication**	**Clade**	**Gene ID**	**Name synonym**	**Validation**	**Publication**
NAC	AT2G46770; AT1G32770; AT3G61910	NST1; NST3/SND1; NST2	Mitsuda et al., 2005, 2007; Zhong et al., 2006, 2007b	C	Zm00001d045463	ZmSWN1	complement *snd1/nst1* At mutant + Oe activates SCW TF	Zhong et al., 2011a
					LOC_Os06g04090	OsSWN1		
					Sobic.007G018100	SbSWN2/SbNSTa		
					Sobic.010G022400	SbSWN1/SbNSTb		
	AT4G28500; AT1G28470	SND2; SND3	Hussey et al., 2011	G	Sobic.003G251800	SbSNDb		
					Sobic.009G231600	SbSNDa		
	AT1G71930	VND7	Kubo et al., 2005	C	LOC_Os08g01330	OsSWN3	complement *snd1/nst1* At mutant + Oe activates SCW TF	Zhong et al., 2011a
					JN634079	ZmSWN3		
					Sobic.007G003000	SbSWN3/SbVND7a		
					Sobic.006G279400	SbSWN2/SbVND7b		
	AT1G12260; AT1G62700; AT5G62380	VND4; VND5; VND6	Ohashi-Ito et al., 2010	C	Sobic.010G002900	SbSWN5/SbVND4-6a		
					Sobic.006G160900	SbSWN6/SbVND4-6c		
					Sobic.004G302400	SbSWN7/SbVND4-6b		
					JN634082	ZmSWN6	Complement *snd1/nst1* At mutant + Oe activates SCW TF	Zhong et al., 2011a
					JN634083	ZmSWN7		
					LOC_Os06g01480	OsSWN7		
	AT2G18060; AT4G36160; AT5G66300	VND1; VND2; VND3	Zhou et al., 2014	C	Sobic.001G316800 Sobic.001G522700	SbVND1-3a SbVND1-3b		
	AT4G29230	NAC075	Endo et al., 2015	G	Sobic.003G035100	SbNAC75a		
					Sobic.006G004100	SbNAC75b		
					Sobic.006G003800	SbNAC75c		
					Sobic.009G071600	SbNAC75d		
					Sobic.009G071500	SbNAC75e		
	AT5G13180	VNI2	Yamaguchi et al., 2010	K	Sobic.002G259600	SbVNI2g		
					Sobic.007G190333	SbVNI2f		
					Sobic.003G409800	SbVNI2e		
					Sobic.003G423200	SbVNI2d		
					Sobic.006G141900	SbVNI2c		
					Sobic.008G094700	SbVNI2b		
					Sobic.005G056300	SbVNI2a		
MYB	AT3G08500; AT5G12870	MYB83; MYB46	Zhong et al., 2007a; McCarthy et al., 2009; Zhong and Ye, 2012	4	Sobic.008G112200	SbMYB83		
					Zm00001d023931	ZmMYB46	Oe in At activates SCW biosynthesis program	Zhong et al., 2011a
					LOC_Os12g33070	OsMYB46		
					Potri.001G267300	PtrMYB3	Oe leads to ectopic deposition of SCW	McCarthy et al., 2010
					Potri.009G061500	PrtMYB20		
					Potri.001G258700	PtrMYB21		Zhong et al., 2013
					Potri.009G053900	PtrMYB2		
					Eucgr.G03385	EgMYB2	Oe in tobacco increases SCW thickness	Goicoechea et al., 2005
	AT1G09540; AT4G01680	MYB61; MYB55	Newman et al., 2004; Romano et al., 2012	4	Sobic.009G036500	SbMYB61a		
					Sobic.003G136600	SbMYB61b		
					LOC_Os01g18240	OsMYB55/61	RNAi plants show abnormal phenotype	Hirano et al., 2013b
	AT1G63910	MYB103	Zhong et al., 2008	4	Sobic.007G039100	SbMYB103		
					LOC_Os08g05520	OsMYB103	RNAi plants show abnormal phenotype	Hirano et al., 2013b
	AT5G16600; AT1G66230	MYB43; MYB20	Zhong et al., 2008	4	Sobic.007G132600	SbMYB20/43a		
					Sobic.002G196100	SbMYB20/43b		
					Sobic.002G196000	SbMYB20/43c		
					Sobic.004G248700	SbMYB20/43d		
					Sobic.010G106601	SbMYB20/43e		
	AT1G16490; AT1G79180	MYB58; MYB63	Zhong et al., 2008; Zhou et al., 2009	1B	Sobic.004G273800	SbMYB60		Scully et al., 2016
					Sobic.006G199800	SbMYB60b		
	AT1G17950; AT1G73410	MYB52; MYB54	Zhong et al., 2008; Zhong and Ye, 2012	12B	Sobic.001G110900	SbMYB52/54		
	AT4G33450	MYB69	Zhong et al., 2008	12B	Sobic.008G063400	SbMYB69a		
					Sobic.005G104800	SbMYB69b		
	AT4G12350; AT4G22680	MYB42, MYB85	Zhong et al., 2008	4	Sobic.002G275500	SbMYB42a		
					Sobic.007G178300	SbMYB42b		
					LOC_Os09g36250	OsMYB42/85	RNAi plants show abnormal phenotype	Hirano et al., 2013b
					Zm00001d032032	ZmMYB167	Oe in Zm lead to increased lignin	Bhatia et al., 2019
	AT4G38620; AT2G16720; AT4G34990	MYB4; MYB7; MYB32	Preston et al., 2004; Mitsuda et al., 2007; Ko et al., 2009	15A	Sobic.007G177100	SbMYB4b		
					Sobic.002G279100	SbMYB4a		
					Eucgr.G01774	EgMYB1	Oe in At reduces SCW thickening	Legay et al., 2010
					Zm00001d053220	ZmMYB42	Oe decreases lignin content + downregulates COMT	Fornalé et al., 2006
					Zm00001d006236	ZmMYB31		

*Validation method and publication are reported, as well.*

### Phylogenetic Analyses of the MYB Transcription Factor Family

Using the same procedure as for the NAC genes, sequences from nine different species were retrieved from Phytozome database to revise the phylogenetic relationships of the MYB family and for the identification of SbMYB genes that could be involved in SCW establishment. One hundred forty-four genes were identified in Arabidopsis, 210 in poplar, 171 in eucalyptus, 102 in barrel medic, 180 in maize, 135 in foxtail millet, 126 in rice, 125 in brome and 135 in sorghum (Supplementary Table S8). To the same extent as for NAC genes, these numbers globally match to the ones previously described for these species. One hundred ninety-seven (Katiyar et al., 2012), 198 (Yanhui et al., 2006), and 133 (Li et al., 2016) MYB genes were identified in Arabidopsis, 191 in poplar (Wilkins et al., 2009),141 in eucalyptus (Soler et al., 2015), 155 in barrel medic (Zhong and Ye, 2015), 157 in maize (Du et al., 2012), 209 in foxtail millet (Muthamilarasan et al., 2014), 233 (Smita et al., 2015), 155 (Katiyar et al., 2012), and 163 (Yanhui et al., 2006) MYB in rice and 122 in brome (Zheng et al., 2017).

Because there is no published MYB phylogeny in *Sorghum bicolor*, we used the most recent study involving Arabidopsis MYB genes (Li et al., 2016) as a base to anchor our multi-species classification. Li et al. (2016) described 133 Arabidopsis genes allocated to fourteen clades (1–14). Clade numbering was rearranged taking into account the position of Arabidopsis genes in our phylogenetic tree. All of the Arabidopsis genes identified in Li et al. (2016) were included in the 144 Arabidopsis MYB genes selected for this study. Most of the clades in the multispecific phylogenic tree were recovered and most of the Arabidopsis genes maintained similar phylogenetic links (Figure 2B, Supplementary Figure S5, and Supplementary Table S8). Clades 2, 4, 5, 7, 8, and 11 were conserved even though clades 5, 8, and 11 lost a few Arabidopsis genes that were allocated to other branches in the tree. Clades 1, 3, 6, 10, and 12 were separated into two subclades each, named A and B. A new clade, clade 15, has been created and is mainly composed of genes from former clades 9 and 13 in addition to other genes originating from other former clades. Former clades 13 and 9 in this new clade 15 are specifically identified as 15A and 15B. A new multi-specific clade named 16 was identified and included mainly Arabidopsis genes not described in Li et al. (2016).

Overall, 23 Arabidopsis genes did not show the same phylogenetical link between our work and Li et al.’s (2016) publication. Eleven new Arabidopsis genes were included in our analysis compared to Li et al. (2016) work. Nine of these genes were allocated to the new clade 16 and to two other clades. These new genes are listed as MYB genes on other databases and 4 are MYB-related (Jin et al., 2017). The 135 SbMYB genes identified are distributed in all of the clades.

Clades 4 and 15 encompassed a large number of genes as a result of the merging of several Arabidopsis subgroups from the tree provided by Dubos et al. (2010). Clade 4 includes subgroups S13 and S16, while clade 15 includes S4, S6, S7, and S15. Clades 1B and 15 contain a small number of monocotyledon genes (33 and 26%, respectively), whereas an average of 42% of monocotyledon sequences was observed over all the clades. This could be explained by diversification events within the two perennial woody species (populus and eucalyptus). Clade 1B from our analysis is composed of subgroups S2 and S3 from Dubos et al. (2010). Genes from subgroup S3 have been identified to be involved in CW biosynthesis. Clade 15 includes Arabidopsis subgroups S4, S5, S6, and S7, all of them involved in phenylpropanoid synthesis regulation (Dubos et al., 2010). On the other hand, we observed an enrichment of monocotyledon sequences in clade 1A (57%). This clade includes genes, from subgroup S1 in Arabidopsis, that have been reported to be involved in abiotic stress responses (Seo et al., 2009; Seo and Park, 2010; Li B. et al., 2019).

Clade 4 includes nine of the 17 MYB genes that have been reported to have a role in SCW establishment. Two of them, AtMYB46 and AtMYB83, are redundant genes in Arabidopsis and in our phylogenetic analysis we found only one ortholog for each of the monocotyledon species (Figure 3C and Table 1). Another example is AtMYB103, which has only one ortholog in monocotyledon species (Figure 3E and Table 1).

In addition to the clade 4, clades 1B, 12, and 15 also contain MYB genes involved in SCW composition (2, 3, and 3 respectively). Among these genes, AtMYB4, a negative regulator of SCW belonging to clade 15, presents at least two orthologs in each of the monocotyledon species (Figure 3D and Table 1).

### Whole Genome Sorghum Transcriptome Co-expression Network

#### Identification of Co-expressed Gene Modules Potentially Involved in Cell Wall Construction

The first step to construct expression network to dissect molecular mechanisms involved in a specific physiological event (here construction of SCW) is to obtain multiple expression data from several conditions and developmental stages when those events take place. In this purpose, identification of the gene co-expression networks has been conducted on the analysis of three field experimental trials. Slightly more than 4.7 billion of raw reads were obtained over these experiments with 1.95, 1.63, and 1.39 billion reads respectively for 2013, 2014 and 2015. On average, 5% of the sequences were discarded after the Fastq cleaning step and 85% were successfully mapped on the V3.1 reference genome (for a total of 3.96 billion reads). After filtering based on the expression level, a total of 20,294 genes were considered for the co-expression analysis. Forty-five modules containing from 32 to 2937 genes were originally detected but only 30, totaling 12422 genes, were kept according to the filter used for the correlation expression threshold. Among these clusters, four clusters contained less than 10 genes, 12 had between 10 and 100 genes, 10 had between 100 and 1000 genes, and the remaining four clusters harbored more than 1000 genes (Supplementary Figure S2B).

**FIGURE 4:**
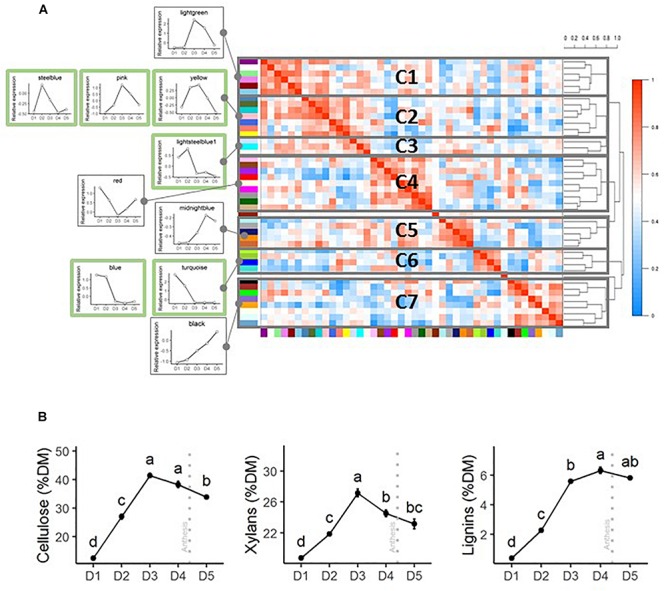
Expression patterns of the co-expression modules identified through the WGCNA analyses and dynamics of accumulation of the main cell wall components. **(A)** Correlation heatmap based on the normalized expression pattern of all the genes of each module. Clustering of the modules in 7 clusters was based on the distance dendrogram provided on the right. The mean gene expression patterns of some of the modules for the 2013 experiment (internode 12 in irrigated conditions) are presented on the left side of the correlation heatmap to illustrate the general patterns of expression along the five developmental stages that have been analyzed. Modules for which enrichments in SCW and general CW GO terms and CW-related enzymes have been detected are framed in green color. Although the presented expression patterns only correspond to the 2013 trial, the correlations and the distances among the modules are based on the expression patterns observed over the three trials. **(B)** Patterns of accumulation of the three main cell wall components in internode 12 of the well-watered treatment of the 2013 trial. Stages with different letters present significantly different mean values. The error bars correspond to the standard errors based on four biological replicates. The anthesis date is indicated by a vertical gray dotted line.

Clustering of the normalized expression pattern of every module shows groups of modules with similar expression patterns (Figure 4A). Seven clusters of modules (C1–C7) were identified. To better illustrate the dynamics of gene expression during internode development within these modules, the expression patterns observed for the internode 12 in the WW treatment of the 2013 trial were used. Cluster C5 presents high expression levels in the very first stage D1. Clusters 1–3 encompass genes that present increases in expression between stages D1–D2 (C1 and C3) and D1–D3 (C2). Clusters 5 and 7 include genes with increasing expression throughout the stages. Cluster C4 presents a decrease in gene expression from D1 to D3 followed by a continuous increase until D5.

A custom list of enzymes annotated as involved in CW establishment was compiled based on previous publications (Supplementary Table S3) and the 30 modules were tested for enrichment in this CW related enzyme list as well as in CW related gene ontology (GO) terms for biological processes (BP) and cellular components (CC) (Table 2 and Supplementary Table S1).

**TABLE 2:** Enrichment analyses of the modules based on a cell wall-related enzyme list and SCW and general CW GO terms (CW GO terms).

**Module**	**Cell wall related enzyme list enrichment (*p*-value)^1^**	**CW GO enrichment^2^**		**Number of genes^3^**
		**BP**	**CC**	
Yellow	2.07^∗^10^–34^	22	1	1616
Blue	8.72^∗^10^–5^	11	2	2159
Turquoise	0.00173	8	0	2855
Pink	0.0093	7	0	283
Steelblue	6.01^∗^10^–5^	6	1	52
Lightsteelblue	1.59^∗^10^–5^	4	2	9
Darkturquoise	0.16^ns^	1	0	38
Orangered	0.33^ns^	2	0	21
Darkgreen	0.49^ns^	2	0	86
Cyan	0.52^ns^	5	0	248
Green	0.98^ns^	3	0	671
Royalblue	1^ns^	3	1	63
Lightgreen	1^ns^	0	1	5
SaddleBrown	1^ns^	0	1	5
Greenyellow	1^ns^	0	1	426

*Only modules with at least one significant enrichment are presented. ^*1*,*ns*^Stands for non-significant enrichment. ^2^Number of CW GO terms [Biological Process (BP) and Cell Compartment (CC)] significantly (*p*-value < 5%) enriched in each module. ^3^Total number of genes in the considered module.*

Six modules, turquoise, yellow, pink, blue, steelblue and lightsteelblue, were significantly enriched in the CW-related enzyme custom list (Table 2 and Supplementary Table S1). The same six modules were also enriched in multiple SCW and general CW related GO terms (CW GO terms) (from 4 to 22 BP GO terms and from 0 to 2 CC GO terms). Six additional modules were enriched in between 1 and 5 BP GO terms related to CW but were not significantly enriched in the custom CW genes (royalblue, green, cyan, darkgreen, orangered, and darkturquoise). Three modules have one enriched CC GO terms related to CW but were not enriched in the custom list of CW genes (saddlebrown, lightgreen, greenyellow) (Supplementary Table S1). Furthermore, two out of these three last modules contain very low numbers of genes. Only the six modules that were enriched both in CW GO terms and genes from the custom list were further investigated (Table 2).

The yellow module presents the highest number of enriched GO terms corresponding to general CW (22) and more specific SCW ontology terms with an overrepresentation of pectin, cellulose, xylan, phenylpropanoid and lignin related genes (Table 3). The pink and blue modules are also enriched in SCW GO terms but more specifically in lignin and phenylpropanoid related genes. The turquoise, steelblue and lightsteelblue modules are enriched in pectin, cellulose, xylans and general CW GO terms (Table 3).

**TABLE 3:** Enrichment of MYB and NAC bait gene subnetworks and WGCNA modules in GO terms related to SCW and general CW (CW GO terms).

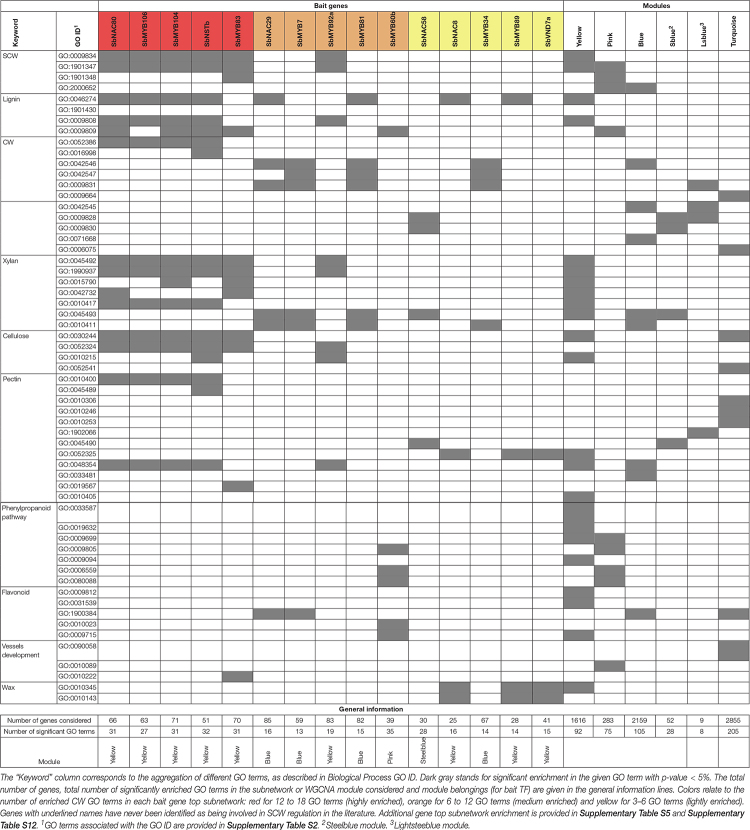

*The “Keyword” column corresponds to the aggregation of different GO terms, as described in Biological Process GO ID. Dark gray stands for significant enrichment in the given GO term with *p*-value < 5%. The total number of genes, total number of significantly enriched GO terms in the subnetwork or WGCNA module considered and module belongings (for bait TF) are given in the general information lines. Colors relate to the number of enriched CW GO terms in each bait gene top subnetwork: red for 12 to 18 GO terms (highly enriched), orange for 6 to 12 GO terms (medium enriched) and yellow for 3–6 GO terms (lightly enriched). Genes with underlined names have never been identified as being involved in SCW regulation in the literature. Additional gene top subnetwork enrichment is provided in Supplementary Table S5 and Supplementary Table S12. ^1^GO terms associated with the GO ID are provided in Supplementary Table S2. ^2^Steelblue module. ^3^Lightsteeblue module.*

In terms of gene expression, the mean expression of genes belonging to yellow and pink modules both within the C2 cluster reached a peak at the D3 stage of the 2013 trial (for internode 12 in the well-irrigated treatment). The maximal expression of other modules enriched in CW GO terms occurs earlier in development. The mean expression of genes belonging to turquoise and blue modules present a maximum expression level at the D1 stage whereas the steelblue and lightsteelblue modules peak at the D2 stage.

Accumulation patterns of SCW components in the internode 12 of the WW of the 2013 field trial are presented in Figure 4B. Xylans and cellulose reach a maximum at the D3 stage, slightly earlier than lignin, a component specific to SCW.

#### Identification of Hub Genes in the Cell Wall Enriched Modules

Within each CW-related module, highly connected genes were identified using the intramodular connectivity parameter. The top 10 genes for each module are reported in Table 4. With the exception of the lightsteelblue module that contains a low number of genes, the 10 top genes are the same, regardless of whether the correlation threshold is considered or not revealing that the genes harboring the largest numbers of connections are also the ones that harbor the highest correlations with the other genes of the module. Some of these top genes are classified as TF according to their GO term (GO:0003700). These TFs are, Sobic.002G260800 which belongs to the ERF protein family, in the steelblue module, Sobic.004G237300, a TCP TF, in the blue module, and Sobic.003G148600 and Sobic.010G080400, which also both belong to the ERF protein family, in the lightsteelblue module.

**TABLE 4:** Top hub genes in the six cell wall-related modules.

**Module**	**Gene ID**	**Putative role (based on *Arabidopsis thaliana* and *Oryza sativa* Phytozome version 12 best hits)**	**CW GO^1^**	**Number of genes in top 10%**	**Number of genes with CW GO terms in top 10%**
Blue	Sobic.001G502900	HR-like lesion-inducing protein-related		216	13
	Sobic.003G011100	VIRB2-interacting protein 1/reticulon-like			
	Sobic.003G223100	Polygaracturonase (pectin lyase-like)			
	Sobic.004G237300	TCP family transcription factor	x		
	Sobic.004G323100	Cysteine rich secretory protein/defense			
	Sobic.004G333500	Proteasome maturation factor UMP1			
	Sobic.006G046200				
	Sobic.007G180200				
	Sobic.008G035400	Stress responsive A/B Barrel Domain			
	Sobic.008G051900	ATPase subunit			
Turquoise	Sobic.001G365266	Zing-finger type protein		283	15
	Sobic.002G279400	Protein of unknown function			
	Sobic.004G063600	Vacuolar import/degradation, Vid27-related protein			
	Sobic.004G269100	PRONE (Plant-specific Rop nucleotide exchanger)	x		
	Sobic.006G124400	ATP binding protein/DNA-directed DNA polymerase			
	Sobic.006G241800	Leucine-rich repeat protein kinase family protein			
	Sobic.007G201900	Protein of unknown function			
	Sobic.009G172950	Minichromosome maintenance (MCM2/3/5) family protein/DNA helicase			
	Sobic.010G077000	Leucine-rich receptor-like protein kinase family protein	x		
	Sobic.010G129700	Microtubule-associated protein 65-2			
Pink	Sobic.001G110900	Homolog of AtMYB52/54	x	29	3
	Sobic.001G131400	Thioesterase superfamily protein			
	Sobic.001G361300	CDPK-related kinase 1			
	Sobic.001G372000	F-box and tubby domain containing protein			
	Sobic.001G482100	Plant protein of unknown function			
	Sobic.002G200700	Protein of unknown function (DUF1666)			
	Sobic.003G035100	Homolog of AtNAC075	x		
	Sobic.003G317500	Lysophosphatidyl acyltransferase 5			
	Sobic.004G102400	RING/FYVE/PHD-type zinc finger family protein			
	Sobic.006G145901	Beta glucosidase 46/monolignol beta-glucoside homolog			
Yellow	Sobic.001G038300	TRICHOME BIREFRINGENCE-LIKE 33		161	31
	Sobic.001G063500	FASCICLIN-like arabinogalactan protein 17 precursor			
	Sobic.002G128800	Protein of unknown function			
	Sobic.002G252000	Chitinase family protein	x		
	Sobic.003G266400	ENTH/ANTH/VHS Traffichink pathway/clathrin assembly protein			
	Sobic.004G111100	GDSL-like Lipase/Acylhydrolase superfamily protein			
	Sobic.004G221300	Glucose-6-phosphate/phosphate and phosphoenolpyruvate antiporter	x		
	Sobic.005G194900	Phosphoserine phosphatase			
	Sobic.008G003600	Plant protein of unknown function			
	Sobic.010G022300	Transmembrane receptor			
Steelblue^2^	Sobic.001G359700	DUF679 domain membrane protein 2		<10	0
	Sobic.002G260800	Integrase-type DNA-binding superfamily protein AP2 domain			
	Sobic.004G310000	Lipase/alpha/beta-Hydrolases superfamily protein			
	Sobic.006G232500	Nuclease			
	Sobic.006G244400	Nuclease			
	Sobic.007G172100	Cysteine protease			
	Sobic.009G257400	Alpha-vacuolar processing enzyme			
	Sobic.010G078200				
	Sobic.010G133866				
	Sobic.010G158200	Protein of unknown function			
Lightsteelblue^3^	*Sobic.001G080100*			<10	2
	*Sobic.001G304201*	*LTPL141 - Protease inhibitor/seed storage/LTP family protein precursor*			
	*Sobic.003G148600*	*Integrase-type DNA-binding superfamily protein AP2 domain*			
	*Sobic.003G432700*	*Curculin-like (mannose-binding) lectin family protein*			
	Sobic.007G146200	Plant invertase/pectin methylesterase inhibitor superfamily	x		
	*Sobic.007G214600*	*Calcium-binding EF-hand family protein*			
	*Sobic.009G152600*	*Pyrophosphorylase 3*			
	Sobic.009G173700	Expansin	x		
	*Sobic.010G080400*	*Integrase-type DNA-binding superfamily protein AP2 domain*			
	*Sobic.007G214400*	*Calcium-binding EF-hand family protein*			

*Hub genes have been identified using the intramodular connectivity. For each module, the top 10 hub genes, their putative roles and their annotation with SCW and general CW GO terms (CW GO terms) are given. In addition, the number of 10% top hub genes in each module and the number of these genes with at least one CW GO term are provided. A detailed list of the top 10% of each module can be found in Supplementary Table S10. ^1^“x” means the gene has at least one CW GO term annotation. ^2^As there are only 52 genes in the steelblue module, top 10% information is equivalent to top 10 genes. ^3^As they are only 9 genes above the correlation threshold in the lightsteelblue module, we report the top 10 genes without considering the correlation threshold. The two genes above the correlation threshold are indicated in upright font.*

There are one additional NAC gene and one additional MYB gene in the top 10 genes of the pink module but they are not annotated with the TF GO term. Sobic.003G035100 (SbNAC75a) is a close homolog of NAC075, a putative regulator of VASCULAR RELATED NAC-DOMAIN PROTEIN 7 (VND7, Supplementary Figure S4B) which is a master regulator of vessel SCW deposition in Arabidopsis (Endo et al., 2015; Fujiwara and Mitsuda, 2016). Sobic.001G110900 is a homolog of AtMYB52/54, another regulator of SCW (Zhong et al., 2008).

The TCP TF in the blue module is annotated as being involved in flavonoid metabolic processes. Other genes have ontologies related to CW (eight in total) and all five modules have at least one of their top 10 genes annotated as CW related, except the steelblue module.

In addition to the top 10 genes, the “top 10% genes” of each of the six CW-related modules were also retrieved for further analyses (Table 4 and Supplementary Table S10). SbVND7a belongs to the top 10% gene list of the yellow module which is the one presenting the most CW-related ontologies annotated genes (31). The blue, turquoise and pink modules “top 10% hub gene lists” are composed of between 5 and 10% of genes with GO terms related to CW. The blue module has neither TF annotated genes nor MYB or NAC genes in the top 10% hub genes, and the turquoise module has three MYB genes, none of which are close homologs of MYB genes validated in other species for their role in SCW regulation. There are also two TFs belonging to the GATA and WRKY TF families in the top 10% hub genes of the pink module.

### Co-expression Networks of NAC and MYB Transcription Factors

Among the 257 SbMYB and SbNAC genes included in the phylogenetic analysis, 83 MYB and 86 NAC genes were discarded because of their low expression levels or because they did not show any strong correlation with other genes (Supplementary Table S11). Among these genes, some sorghum homologs of putative key genes based on previous publications were not considered according to their low expression levels (SbSWN6/SbVND4-6c, SbSWN2/SbVND7b, SbVND1-3a,b, SbNAC75c,d,e, SbVNI2a,d,c and SbMYB20/43c, SbMYB69a,b) or a correlation with the other genes lower than the selected threshold (SbMYB20/43a, SbMYB42b, SbMYB4b, SbNAC75b, SbVNI2f and SbMYB60).

**FIGURE 5:**
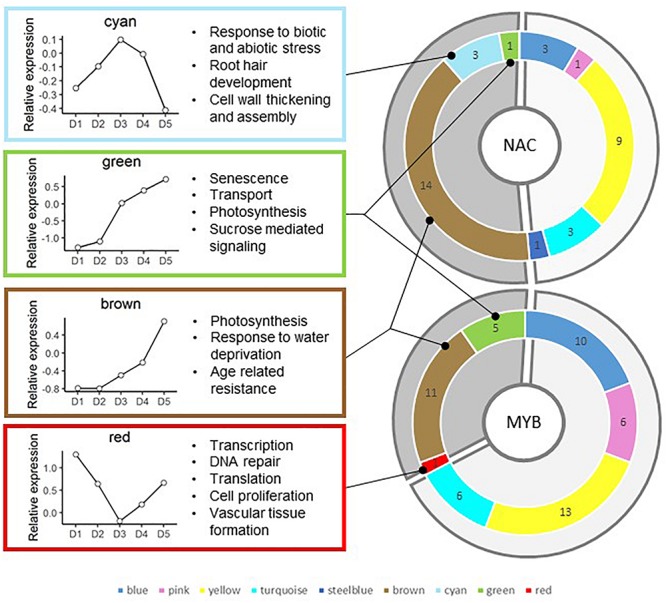
Distribution and number of sorghum NAC and MYB genes in the different modules. Modules enriched in SCW and general CW-related GO terms are highlighted in clear gray. Modules not directly related to cell wall establishment (i.e., brown, cyan, green, and red) are highlighted in darker gray. The main results of the GO term enrichment analysis are provided in the left boxes in addition to their average expression profile in the 2013 field trial experiment.

Most of the 52 MYB and 36 NAC genes included in the co-expression network analysis belong to the brown (11 MYB and 14 NAC), blue (10 MYB and 3 NAC) and yellow (13 MYB and 9 NAC) modules (Figure 5). The remaining MYB and NAC genes are found in the turquoise, steelblue, and pink modules, but also in the red, green, and cyan modules. There is no MYB or NAC gene in the lightsteelblue that only contains nine genes.

We used the 88 MYB and NAC genes as bait genes to identify highly co-expressed genes. We chose to extract the list of the top 15 correlated genes with the guide-gene and the top 15 correlated genes of each of those top 15 genes. Theoretically, the list of top subnetwork correlated genes is composed of 241 genes. The lists of top gene subnetworks obtained contained between 22 and 102 genes with an average number of 65.8 genes (Supplementary Table S4). We characterized the GO terms enrichment of these lists and focused especially on CW GO terms (Supplementary Tables S4, S5).

Apart from one gene in the green module, all 30 MYB and 14 NAC genes whose top subnetwork is enriched in GO terms related to CW belong to the module presenting CW and SCW enrichment (either in GO terms or CW related genes). There are 22 TF top subnetworks enriched in CW GO terms in the yellow module, 10 in the blue module, seven in the pink module, three in the turquoise module and one in the steelblue module (Supplementary Table S11). We further investigated TFs whose top subnetworks are enriched in three to 18 GO terms related to CW (39 genes) and classified them into three categories of enrichment: “high” between 12 and 18, “medium” between 6 and 12 and “light” between 3 and 6. All 19 highly enriched TF top subnetworks belong to the yellow module, two medium enriched subnetworks belong to the yellow module, five belong to the blue module, five belong to the pink module and three lightly enriched subnetworks belong to the yellow module, three belong to the blue module, one belongs to the pink module, one belongs to the steeblue module and one belongs to the turquoise module.

Top subnetwork enrichments of every SbMYB and SbNAC ortholog or close paralog of validated MYB and NAC in other species were analyzed. In most cases, homologs’ top subnetworks were enriched in GO terms related to CW or SCW (Tables 1, 4 and Supplementary Table S11).

The top subnetworks of SbNSTb and SbNSTa and SbSNDb and SbSNDa show high enrichments in GO terms specific to lignin biosynthesis and more general SCW ontologies (Supplementary Table S12). The homologs of the Arabidopsis VND1 to VND6 (Supplementary Figure S4B), which are involved in specific vessel SCW deposition, either did not pass the selected thresholds for the expression and correlation levels or presented top subnetworks (SbVND4-6a in the turquoise module and SbVND4-6b in the blue module) only lightly or not enriched in CW GO terms. The top subnetwork of the only one of the two sorghum orthologs of Arabidopsis VND7 that met our selection criteria, SbVND7a, is lightly enriched in ontologies related to CW waxes (suberin, cutin), polyphenolic compounds and pectin.

The top subnetwork of SbVNI2g, one of the seven orthologs of AtVNI2 (Supplementary Figure S4A), a regulator of SCW in Arabidopsis interacting with VND7, is the most highly enriched in CW GO terms along with SbSNDa top subnetwork.

The other major regulator of SCW deposition in Arabidopsis, maize and rice is MYB46 which is functionally redundant with its paralog AtMYB83. In sorghum, one direct ortholog of the redundant AtMYB46 and AtMYB83, SbMYB83 (Figure 3C) was identified belonging to the yellow module. The top subnetwork of this gene is highly enriched in 12 GO terms related to general CW and SCW GO terms (xylan, and cellulose) and SCW-specific GO terms (Table 3).

SbMYB60 and its paralog SbMYB60b are orthologs of AtMYB58 and AtMYB63. SbMYB60 has been functionally validated for its role in the sorghum lignin pathway (Scully et al., 2016, 2017) but it did not pass our correlation threshold. SbMYB60b is allocated to the pink module, and its top subnetwork is medium enriched in one GO term related to lignin, three GO terms related to phenylpropanoid and two GO terms related to flavonoid biosynthesis.

Downstream SbMYB homolog genes of SCW regulators (Figure 1) are also highly enriched in CW GO terms. The top subnetworks of SbMYB4a, an ortholog of the SCW repressor gene group encompassing AtMYB4, AtMYB7, AtMYB32, and SbMYB61b are both enriched respectively in 18 CW GO terms. And the top subnetwork of SbMYB103 included 17 CW GO terms (Supplementary Table S5).

We also detected enrichment in the top subnetworks of SbMYB and SbNAC genes whose orthologs were not annotated as involved in SCW regulation in any other species. Based on our classification of GO-enrichment level, we identified four SbMYB and one SbNAC highly enriched in CW GO terms (SbMYB17, SbMYB40, SbMYB104, SbMYB106, and SbNAC80); nine SbMYB and one SbNAC moderately enriched (SbMYB7, SbMYB43, SbMYB53, SbMYB76, SbMYB81, SbMYB92, SbMYB92a, b, and c, and SbNAC29); and three SbMYB and three SbNAC genes lightly enriched (SbMYB34, SbMYB55, SbMYB89, SbNAC16, SbNAC28 and SbNAC58) (Table 3 and Supplementary Table S5).

All highly enriched TF top subnetworks allocated to the yellow module are enriched in GO term “plant-type SCW biogenesis,” as well as one lignin process GO term (“metabolic,” “catabolic,” “biosynthesis”), “xylan biosynthetic process” and “cellulose biosynthetic process.”

The nine medium-enriched TF top subnetworks are allocated to the blue, pink and yellow modules, and only two of them are enriched in SCW GO terms. The four in the pink module are enriched in flavonoid and phenylpropanoid GO terms.

None of the lightly enriched TF top subnetworks, which are allocated to the blue, steelblue and yellow modules, is enriched in SCW GO terms. SbNAC8, SbMYB89 and SbVND7a, all in the yellow module, are enriched in wax GO terms.

**FIGURE 6:**
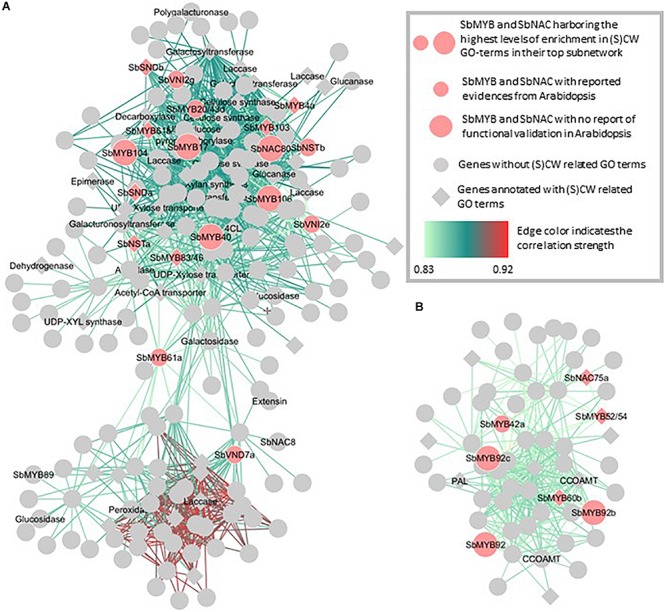
Focused representations of the cell wall transcription factors co-expression networks from the yellow **(A)** and pink **(B)** modules. The top co-expression subnetworks of sorghum orthologs of MYB and NAC TFs either functionally validated in Arabidopsis for their role in SCW (small light orange circles) or of new SbMYB and SbNAC TFs (large light orange circles) whose subnetworks are enriched in cell wall-related GO terms but for which no evidence have been reported in Arabidopsis to date are presented here. The new SbTFs reported in this figure are the ones with the most SCW and CW related GO terms (CW GO terms) enriched top subnetworks (highly enriched in the yellow module and medium enriched in the pink module). Genes represented by squares correspond to genes annotated with at least one CW GO terms. The names of the MYB and NAC TFs are indicated on the figure together with the names of the enzymes belonging to the list of cell wall structural genes aggregated from the literature. Correlation strengths between the genes are represented using a color gradient from light green (lower correlation) to red (higher correlation).

In the yellow and pink cell wall related modules in Figure 6, the significant contributions of key regulators already highlighted in *Arabidopsis thaliana* together with specific sorghum MYB and NAC were identified. In the yellow module (Figure 6A) the coordinated expressions of 13 MYB and NAC genes for which involvement in cell wall building had already been reported in Arabidopsis together with 5 additional MYB and NAC TF (with cell wall enriched subnetworks) were observed. Similarly in the pink module, the putative key roles of the homologs of AtNAC75, AtMYB58/63, AtMYB52/54, and AtMYB42 (see Figure 1 in which their roles on cell wall establishment regulation are presented) have been detected together with the coordinated actions of three paralogs of SbMYB92 (for which no functional role linked to the cell wall has been yet reported in Arabidopsis). To reinforce the key roles of these TF, it is interesting to note that for both modules, several genes encoding structural enzymes involved in cell wall establishment and deposition are amongst the highest co-expressed genes with these TFs. It is also interesting to emphasize that 138 and 44 genes in the yellow and pink modules subnetworks have never been related to the SCW synthesis process in the literature to date (Figures 6A,B). The key TF s identified through these targeted enrichment analyses for all the NAC and MYB sorghum genes are available in the Supplementary Table S11. The homologs of Arabidopsis whose functions in sorghum seem to be concordant in sorghum and Arabidopsis are indicated together with the new sorghum TFs of potential interest.

## Discussion

An in-depth understanding of the regulation of stem SCW establishment in cereal crops will contribute to enhancing their adaptation to the expectations of the producers and users and monitoring their adaptation to future environmental constraints.

Ontogenic evolution of the biochemical composition of the sorghum stems has been described previously. Lignocellulosic component accumulations were described from six days before anthesis to the plant physiological maturity (53 days post anthesis), underlying an accumulation of these components before anthesis (McKinley et al., 2016). Characterization of earlier internode developmental stages (i.e., long before anthesis) showed that cellulose and hemicellulose accumulations occur in the first stages of internode development (between 0 and 200°C of thermal time cumulated since their elongation’s initiation) whereas lignin accumulation extends up to 400°C after elongation’s initiation (Perrier et al., 2017). Internode development was also characterized at the anatomical level allowing the identification of the cell division, cell expansion, and CW establishment steps (Kebrom et al., 2017; Perrier et al., 2017).

Transcriptomic analyses aiming to identify the genes involved in internode development were performed recently. First, a global sorghum transcriptome atlas was developed taking advantage, among other tissues, of different stem components (internode, pith, and rind) showing that these organs and tissues shared a general transcriptomic profile but also that they diverged significantly from the other samples analyzed (Shakoor et al., 2014). A more specific focus on CW-related genes in sorghum revealed tissue specific expression patterns and responses to abiotic stress constraints (Rai et al., 2016). However, these analyses were based only on different seedling organs, and a more detailed study of the kinetics of internode development was needed. The Mullet’s group then made significant contributions to the understanding of internode development and CW deposition. First, analysis of the evolution of the 10^th^ internode’s transcriptome over eight different stages, helped to elucidate the expression patterns of structural genes involved in the different CW component deposition (McKinley et al., 2016). Then, the specific elongation steps of the internode development were described through the analysis of the four apical internode levels (Kebrom et al., 2017). Although previous publications focused their attention on structural genes, this last study also provided a first appraisal of the contribution of TF s to internode elongation and CW deposition.

According to the previous knowledge available on biomass composition establishment in sorghum, the objectives of the present study were to contribute specifically to a better understanding of the SCW establishment and its regulation in sorghum, with a detailed focus on the contribution of the MYB and NAC TF families. To achieve these goals, we first performed gene co-expression network analyses based on an extensive internode sampling scheme over three field trials. Indeed, the construction of co-expression networks has recently emerged as a powerful method to explore high throughput expression datasets and to dissect partially unknown physiological mechanisms (Higashi and Saito, 2013), including SCW establishment (Ruprecht et al., 2011; Hirano et al., 2013a; Hu et al., 2017; Sibout et al., 2017). We therefore updated the phylogenies of MYB and NAC TF families from nine different species to accurately describe the structures of these gene families in sorghum, and merged these two approaches to contribute to the identification of the main regulators of SCW establishment.

### Identification of Co-expression Gene Networks Contributing to the SCW Establishment

Co-expression gene network analysis taking advantage of multiple internode levels harvested at different developmental stages during three field trials allowed the identification of groups of genes sharing the same expression patterns. Among the six groups of genes consistently enriched in CW GO terms and CW related genes (Table 3 and Supplementary Table S9), two gene co-expression networks harboring several clues of involvement in SCW establishment were identified.

**FIGURE 7:**
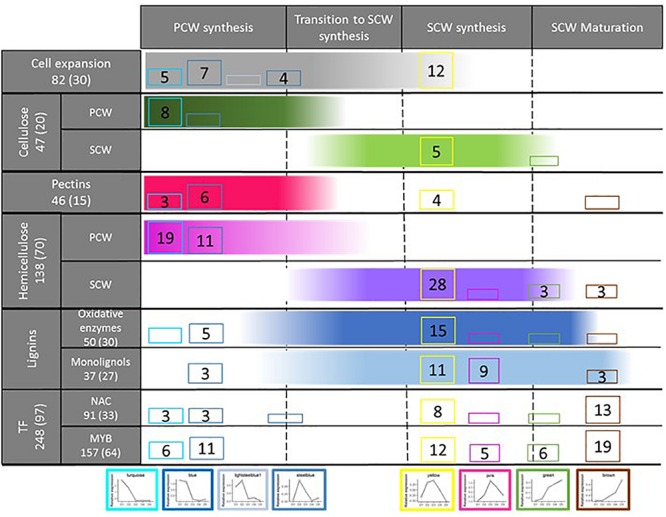
Re-wiring of transcriptome regulation toward SCW establishment in sorghum. The total number of sorghum genes corresponding to the different classes considered in the left column are provided (these numbers are based on the literature review, see Supplementary Table S3). In addition, the total numbers of sorghum genes detected in the modules presented at the bottom of this figure are provided between parentheses. For each module (bottom of the figure), cell wall establishment step (top of the figure) and gene class (left column) considered, the numbers of genes detected through the co-expression network analysis are provided. The color gradients provided for each cell wall establishment step (columns) indicate the most likely timing of expression of the involved genes. Only modules with more than 2 genes for at least one cell wall establishment step are presented. The absence of gene numbers together with the presence of the module color indicates that 1 or 2 genes have been detected. The absence of cluster color indicates that no genes corresponding to the establishment step considered was detected. The structure of this figure has been strongly inspired by Figure 2 of Meents et al. (2018).

These two modules (yellow and pink) present consistent enrichments in SCW GO terms and CW related genes (Figure 7) and expression peaks synchronous with the accumulation patterns of lignin, cellulose and hemicellulose components. The memberships of the three SCW-specific cellulose synthase genes that have been identified in Arabidopsis and validated in rice (in the yellow module), and of two *bmr* genes previously cloned in sorghum (in the pink module: *bmr*12 - Sobic.007G047300 and *bmr*2 – Sobic.004G062500) support the specific relationships of these modules to SCW establishment. These two gene networks are also significantly enriched in laccase, peroxidase, and monolignol biosynthesis genes. In addition, using the 480 genes differentially expressed in the leaves and stalks of CCoAOMT over-expressing lines compared to a wild genotype (Tetreault et al., 2018), we observed that the yellow module encompass the largest number of these genes in comparison with the others modules (49 among the 210 that are present in the 30 co-expression networks that have been analyzed). Meanwhile, taking advantage of the list of 36 genes identified as being co-expressed with SbMYB60 (Scully et al., 2016), we also observed that the yellow module aggregates most of them (17 out of 36).

It is also interesting to mention that consistently with the observation of high expression levels of a small group of cyclin genes (involved in the cell cycle) in mature internodes (Kebrom et al., 2017), expansin genes were also detected in the yellow module, supporting the maintenance of a potential for cell division and elongation in well-developed internodes, probably to allow diameter growth.

These results re-inforce the pioneering works performed by the Mullet’s group (McKinley et al., 2016; Kebrom et al., 2017) through the identification of co-expression gene networks specifically involved in SCW establishment. These findings also open the way toward the identification of genes that do not directly correspond to already known structural SCW-related genes. It is particularly interesting to note that 92.9% (yellow) and 95.8% (pink) of the genes of these SCW modules do not correspond to previously identified CW structural genes (655 CW related genes were identified through a bibliographic review: Supplementary Table S3), giving rise to potential characterization of new structural key players, as well as TFs.

Before presenting the analysis of the regulatory actors potentially contributing to the establishment of the SCW, it is relevant to mention that this work also allowed the identification of four gene networks exhibiting expression patterns rather compatible with an involvement in internode elongation and primary cell wall (PCW) establishment. The two gene co-expression networks harboring the earliest expression peaks (turquoise and blue modules) are significantly enriched in expansins and CW protein genes that are known to be involved in cell elongation (McKinley et al., 2016; Kebrom et al., 2017) (Figure 7) and also contain a great share of the genes involved in pectin, and hemicellulose synthesis that are known to be integrated into the PCWs. GO-enrichment analyses confirmed these specific roles with enrichment in microtubule, pectin, cellulose and xylan GO terms. A few members of the peroxidase and laccase gene classes are also included in these gene networks. As mentioned by Meents et al. (2018), these oxidative enzymes can also act in the early stages of CW establishment. Two other gene networks (lightsteelblue and steelblue) exhibiting slightly delayed expression peaks and also enriched in expansin genes, plant-type CW loosening and pectin associated GO terms were also identified.

### Updated Classifications of the MYB and NAC Transcription Factor Superfamilies: A Necessary Tool to Disentangle SCW Establishment Master Regulators

High expression levels of most of the MYB and NAC genes analyzed by Kebrom et al. (2017) were observed in the older internodes. However, due to the lack of clear phylogenetic frameworks for these two TF families in sorghum, no further interpretations of the observed expression patterns were performed by these authors. To clarify the phylogenic relationships between the different SbMYB and SbNAC genes and provide new insights regarding their evolution compared to Arabidopsis, other dicotyledons and monocotyledon species, we aggregated the MYB and NAC protein sequences from nine different species.

The MYB family, which is one of the largest TF families in plants, has been described in various species such as Arabidopsis, rice, maize and tomato (Dubos et al., 2010; Du et al., 2012; Katiyar et al., 2012; Li et al., 2016) and their roles in controlling different biological processes have been recently reviewed (Ambawat et al., 2013). The current investigation unveiled 135 members in the MYB family of sorghum. Simultaneous phylogenetic analysis of the MYB genes including sorghum and eight additional species allowed the identification of 19 clades. As recently observed in potato (Li X. et al., 2019), a large congruence between our multispecies MYB phylogeny and the ones described in Arabidopsis (Dubos et al., 2010) and tomato (Li et al., 2016) was observed (Supplementary Table S8). With the exception of the clade 3 (subgroup S12 in Dubos et al., 2010) which exclusively contains Arabidopsis members, sequences from dicotyledons and monocotyledons were detected for all the clades, suggesting an expansion of the MYB families before the monocot-dicot divergence.

A recent phylogeny analysis of SbNAC genes reported the existence of 13 NAC clades in sorghum (Sanjari et al., 2019). Our complementary analyses allowed, through the integration of NAC sequences from nine different species, to identify an additional clade (P) and split one of them (L clade) into two subclades (L1 and L2). Only the new P clade identified contains exclusively dicotyledon genes with large multigene families detected in populus and eucalyptus indicating, as for the MYB family, that diversification of the NAC family occurred prior to the monocot-dicot divergence.

Among the different subgroups of NAC and MYB that have been functionally characterized, some specifically regulate plant SCW construction. The roles of thirteen NAC genes in different steps of the developmental process of vascular plant SCWs have been validated to date (Table 1). Most of these genes (10) belong to the C clade described by Sanjari et al. (2019), three are included in the G clade and one belongs to the K clade (Supplementary Table S7).

For the MYB family, 17 TFs have been identified for their impact on SCW establishment (Table 1). According to the review of Dubos et al. (2010), these TFs belong to the subgroups S13 which is equivalent to our clade 4 (two genes and seven others with significant phylogenetic similarity with this subgroup), S4-Clade 15 (three genes), S21-Clade12 (three genes), and S3-Clade1B (two genes).

According to functional redundancies reported for the MYB and NAC genes belonging to the same subgroups/clades in various species, our expectation is that this information will be useful to predict gene function in species currently lacking of in-depth molecular characterizations.

### Most of the Key Regulators of SCW Establishment Seem to Be Convergent Between Arabidopsis and Sorghum

A triple level of information was acquired (i.e., phylogeny, expression profile and function of the genes present in their co-expression network neighborhood i.e., “top subnetwork”) for all the MYB and NAC genes present in the sorghum genome. Overall, the MYB and NAC analyses revealed that for most of the clades containing Arabidopsis TFs with evidence of implication in SCW establishment, homolog sorghum genes harbored expression patterns consistent with the same functions.

**FIGURE 8:**
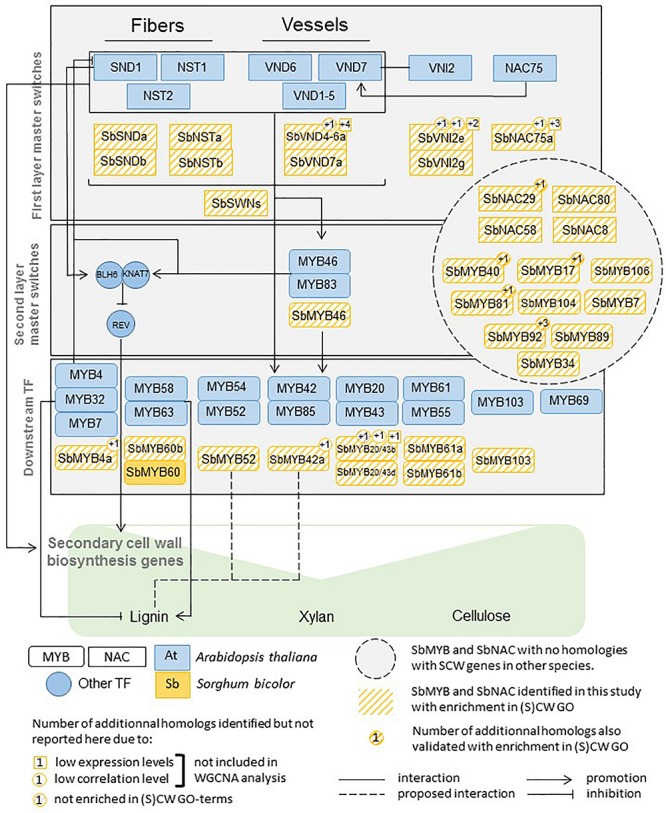
MYB, NAC, and other regulators of SCW biosynthesis functionally validated in Arabidopsis and their sorghum homologs validated in this study using co-expression gene network analysis.

Indeed, starting from the first layer master switches and going down to the downstream TFs (Figure 1) for which functional evidence has been provided in Arabidopsis and a few other species, we managed to identify some sorghum family members mimicking the roles previously identified. A synthesis of these results is provided in Figure 8.

Arabidopsis NST1 to NST3/SND1 are the master regulator switches of SCW construction in Arabidopsis. The two homologs identified in sorghum belong to the SCW-oriented expression module (yellow), and both genes present co-expression networks highly enriched in SCW genes and GO terms. This module also hosts the three SCW-specific cellulose synthases along with other enzymes involved in lignin biosynthesis and polymerization (4CL, laccases). These observations are also consistent with the results reported in maize and rice (Table 1) and, more recently in switchgrass (Rao et al., 2019). In addition to the fiber specific master regulator switches (NSTs), vessel specific TFs of key importance have been also identified. We showed that one ortholog of VND7 (Sobic.007G003000/SbVND7a, Supplementary Figure S4B) that also belongs to the yellow module, in addition to presenting an enrichment in wax related GO terms also harbors a top subnetwork containing 5 structural CW genes and is likely to contribute to the regulation (or at least make part of the top subnetworks) of 4 other downstream MYB genes [SbMYB83 (Sobic.008g112200), SbMYB61a (Sobic.009g036500), Sobic.009g148100, and Sobic.003g270300] (Supplementary Table S4). The absence of any expression for the homologs of AtVND1/2/3 is consistent with the recent findings of Tan et al. (2018) who suggested a specific role of these master regulators switches during seedling development. These results are also consistent with the high expression levels detected for these two genes in the roots and shoots of seedling samples (Morokoshi database^10^).

The second layer of master regulators of SCW building in Arabidopsis involves the redundant AtMYB46 and AtMYB83. The single sorghum ortholog of these two genes belongs to the yellow “SCW-related module” and its top subnetwork is also highly enriched in CW GO terms. In addition, this gene is also a member of the top subnetwork of SbMYB61a, a homolog of AtMYB61, a downstream TF in Arabidopsis (Figure 8).

For the downstream TF groups already validated in Arabidopsis, we reported, among others, clear evidence for the AtMYB103 which has also been validated in rice (Hirano et al., 2013b) (see Supplementary Table S11 and Figure 8 for a complete synthesis of the results). In sorghum, a single ortholog has been identified (SbMYB103), and its expression pattern (it belongs to the pink module) and co-expressed partners are strongly in favor of conservation of its role across deep phylogenetic relationships.

In addition to providing strong evidence of functional conservation across species at the gene level [as also observed by Rao et al. (2019) in their comparisons of switchgrass and Arabidopsis], the integration of phylogenetic and gene-co-expression analyses also allowed the identification of NAC and MYB clades harboring different functional behaviors. Indeed, genes belonging to the NAC clade C and MYB clades 1B, 4, 11, and 12 might contribute either to the primary or to the secondary CW construction, whereas the NAC clade G and the MYB clade 15 hosted genes that target SCW establishment more specifically.

The NAC clade C contains 13 sorghum genes, three of which correspond to the previously mentioned orthologs of NST1/2/3 and VND7 that are SCW linked. This clade also encompasses two sorghum orthologs of AtVND4/5/6 (Sobic.010G002900, Sobic.004G302400) which are, based on the set of samples that have been characterized in this study (i.e., internodes at different developmental stages), specifically expressed in the first steps of CW development (elongation and cellulose/hemicellulose/pectin synthesis), on the contrary to their Arabidopsis homologs involved in SCW formation (Yamaguchi et al., 2008).

The same patterns were observed for the MYB clade 1B. This clade includes, on one side SbMYB60b, which is SCW-related (as also shown for its paralog SbMYB60 by Scully et al., 2016), and SbMYB7, which is PCW-related (a member of the blue module with expansins, pectate and pectin lyase and several xyloglucan endotransglucosylases/hydrolases genes co-expressed). The MYB clades 4, 11 and 12 present the same varieties of expression patterns and top subnetwork enrichments (Supplementary Tables S4, S5).

In contrast, SCW-related clades were identified. The NAC clade G, which contains homologs or closely related genes of AtNAC075, exclusively contains genes with evidence for a role in SCW establishment. The same was reported for the MYB clade 15, which contains, among other genes, homologs of AtMYB4 (which has been validated as a lignin repressor), and is oriented toward SCWs.

In addition to the MYB and NAC genes, the co-expression network analyses also allowed the identification of the sorghum orthologs of the WRKY12 and KNAT7 Arabidopsis genes, which are key players in SCW construction, suggesting a similar role in sorghum. These two TFs are among the hub genes of the pink module that is linked to the SCW establishment. For WRKY12, these results are consistent with those previously reported in grasses (Wang et al., 2010; Li et al., 2012; Gallego-Giraldo et al., 2016) and with the recent results reported in switchgrass (Rao et al., 2019), who underlined the potential repressor role on lignin biosynthesis of this gene.

Overall, we observed a relatively good prediction ability of the function of the MYB, NAC and of a few other TF homologs of genes previously validated in Arabidopsis and other monocotyledon species.

### Specificities of SCW Establishment in Sorghum (and Other Cereals) in Comparison to *Arabidopsis thaliana*

A few divergences between sorghum and the model plant *Arabidopsis thaliana* were also identified. One of the most striking divergence between sorghum and Arabidopsis concerned the lack of detection of expression for the homologs (SbMYB69a and SbMYB69b) of AtMYB69 which has been suspected to be a key player in the SND1/NST1-mediated transcriptional regulation of secondary wall synthesis in Arabidopsis (Zhong et al., 2008). Nevertheless, this result is consistent with those reported in switchgrass in which a lack of expression of the two homolog genes of AtMYB69 was also observed (Zhao and Bartley, 2014). In this case, the ontogenic regulation hypothesis reported by Tan et al. (2018) for VND1/2/3 will probably not provide a relevant explanation, as seedling tissues were also available in the switchgrass analyses. Another divergence with Arabidopsis is the potential minor role of the sorghum homologs of VND7 in comparison to other primary and secondary master switches. Although VND7 has been demonstrated to directly and/or indirectly upregulate many genes involved in a wide range of processes in xylem vessel differentiation in Arabidopsis (Yamaguchi et al., 2011), our results in sorghum are considerably less clear. Indeed, although five structural cell wall genes are included in the top subnetwork of SbVND7a only an enrichment of the co-expression network of VND7 in wax biosynthesis and pectin genes was detected, surprisingly. However, divergence in SCW and waxes biosynthesis regulation has already been detected between mono- and dicotyledon plants. By the way, OsSHN is tightly co-expressed with SCW genes in rice, whereas its closest Arabidopsis homolog regulates waxes deposition in Arabidopsis but has a SCW regulation role when overexpressed in rice (Aharoni et al., 2004; Ambavaram et al., 2011). Before rejecting the role of these MYB and NAC TFs in SCW establishment in grasses, complementary analyses at the tissue or group of cell levels will be required.

We also identified 2 NAC (both in the yellow module) and 10 MYB genes (seven in the yellow and three in the pink module) for which potential roles in SCW construction can be proposed. These TFs are not close homologs of previously characterized SCW related Arabidopsis TF (Supplementary Table S13). Four out of these 12 genes do not belong to clades previously identified as being involved in SCW regulation. These results suggest specificities in secondary CW regulation in sorghum and grasses.

Although an in-depth analysis of these new SCW-related TF remains to be performed, it is interesting to point out that the unique rice homologs (LOC_Os05g35500 and LOC_Os12g07640) of the sorghum genes SbMYB92a (Sobic.009g148100), SbMYB92 (Sobic.008g055800), SbMYB92b (Sobic.005g062000), and SbMYB92c (Sobic.008g055700) that belong to the pink module have also been identified by Hirano et al. (2013a) as being linked to SCW establishment through a similar co-expression network analyses.

To sum up, in addition to Kebrom et al. (2017) who identified a common pattern of expression for all the NAC and MYB gene detected during the elongation phase of the internode, we identified a wider diversity of expression patterns allowing to highlight the ones probably contributing specifically to the SCW establishment. Considering the confirmed and new information obtained in our study, we proposed a new scheme for SCW establishment regulation in sorghum (Figure 8).

### Investigating the Natural Nucleotide Diversity of the NAC and MYB Genes

In sorghum, the impacts of the nucleotide variability of MYB and NAC TFs on biomass composition have been exclusively reported on two occasions. A first link was reported between SbNSTb and the saccharification yield through a GWAS analysis (Wang et al., 2013). Our results are in accordance with this observation, as this gene belongs to the yellow module which is related to the deposition of the SCW and presents a co-expressed gene network enriched in CW genes mainly related to cellulose and hemicellulose synthesis (Supplementary Table S12). The second report concerns the NAC gene underlying the D locus (Sobic.006G147400), which control the stem juiciness. It is hypothesized that this gene modulates the proportion of aerenchyma in the middle part of the sorghum stems (Casto et al., 2018). From our perspective, this NAC gene belongs to the B clade and does not present evidence for a potential role in SCW deposition as we did not detect any strong co-regulation with SCW-related genes.

Although previous studies aiming to link the genetic variability of stem SCW composition with the natural nucleotide diversity available in sorghum failed to provide a clear genomic atlas of the key genes controlling the genetic determinism of these traits (with the exception of NST1, Wang et al., 2013), we believe that strategies aiming at assessing the genetic diversity of the MYB and NAC genes that we identified in combination with accurate tissue-based methods should enable the sorghum community to tackle this challenge.

## Conclusion

Vegetative parts and biomass composition are important factors of plant fitness, enabling plant support and carbohydrate storage among others. Sorghum biomass is also key for several agricultural uses from feed to energy production. Unveiling the mechanisms of SCW deposition contributes to a better understanding of SCW regulation toward the development of more efficient breeding schemes. Phylogenetic analysis of MYB and NAC TFs provided a global overview of these gene families over nine monocotyledon and dicotyledon species. Gene co-expression networks involved in the different steps of SCW establishment were also identified. Taking advantage of these two approaches, we identified MYB and NAC genes that are likely to be involved in SCW biosynthesis in sorghum. We identified clades of genes that may be whether specific of SCW or involved in both primary and secondary CW establishment. We identified strong convergences between Arabidopsis, sorghum and other species and shed light on particular divergences that warrant further elucidation. We also identified new sorghum MYB and NAC genes not validated to date in Arabidopsis for which convergences were observed with other monocotyledon species in a few cases.

Although functional validation is the gold standard toward the validation of gene function, complementary approaches are also relevant to improve the identification of the most relevant genes to use in future breeding schemes. Assessment of the expression differences of genotypes harboring different patterns of accumulation (Li Y. et al., 2019; Luquet et al., 2019) and in different environmental conditions (Perrier et al., 2017; Luquet et al., 2019; Virlouvet et al., 2019) would probably allow to refine our choices to maximize their transposition to breeding programs in the future. In addition, it would be interesting to increase the resolution of the transcriptomic analyses through the analysis of expression of specific groups of cells (e.g., parenchyma vs. vascular bundles…) and short time frame sampling. Combining these physiological approaches with analysis of the patterns of nucleotide diversity of these genes may serve to narrow down the list of candidates to track in the future.

## Data Availability Statement

The datasets generated for this study can be found in the https://www.ncbi.nlm.nih.gov/sra (bioproject PRJNA560153).

## Author Contributions

DP, DL, and LR designed the experiments and obtained the funding. SR established and followed the three field experiments and coordinated the internode samplings. AB, ER, and NoT produced the transcriptomic datasets and performed the first analyses on these. J-FD and SB contributed to the comparative genomic analyses. LB and DB produced the biochemical information. LH, DP, and NaT performed the data analysis presented in the manuscript. LH, NaT, and DP wrote the first version of the manuscript that was then enriched by all the co-authors and leaded the editing. LH, NaT, and DP have primary responsibilities for the final content of the manuscript.

## Conflict of Interest

The authors declare that the research was conducted in the absence of any commercial or financial relationships that could be construed as a potential conflict of interest.

## Footnotes

1 http://planttfdb.cbi.pku.edu.cn/

2 https://phylogeny.southgreen.fr/Hennetetal/FigureS5MYBtree_rep.svg and https://phylogeny.southgreen.fr/Hennetetal/FigureS3NACtree_rep.svg

3 www.euralis.fr/

4 https://www.semencesdeprovence.com/

5 https://www.genotoul.fr/

6 https://github.com/SouthGreenPlatform/arcad-hts

7 https://github.com/gpertea/gffcompare

8 ftp://ftp.psb.ugent.be/pub/plaza/plaza_public_monocots_04/GO/

9 http://www.cropontology.org/ontology/CO_324/Sorghum

10 http://sorghum.riken.jp/morokoshi/Home.html
