# Supplementary figures and images for: Transcriptional Regulation of Sorghum Stem Composition: Key Players Identified Through Co-expression Gene Network and Comparative Genomics Analyses
Source: Front Plant Sci. 2020 Mar 3;11:224. doi: 10.3389/fpls.2020.00224 (PMC7064007; doi:10.3389/fpls.2020.00224)

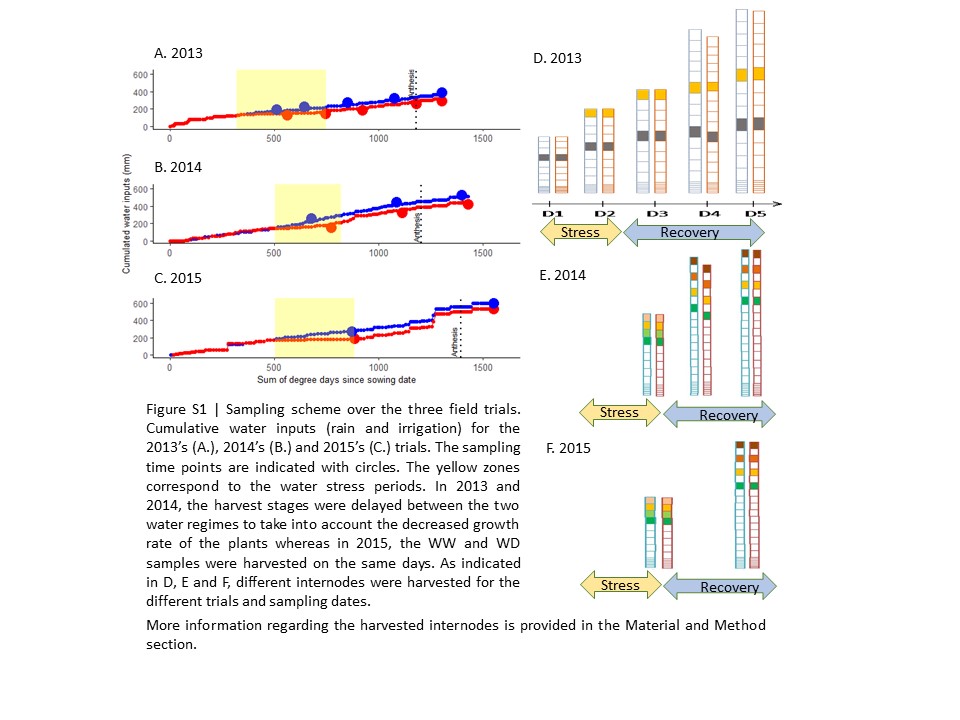

Supplement: Supplementary file 1 [file Image_1.jpg]

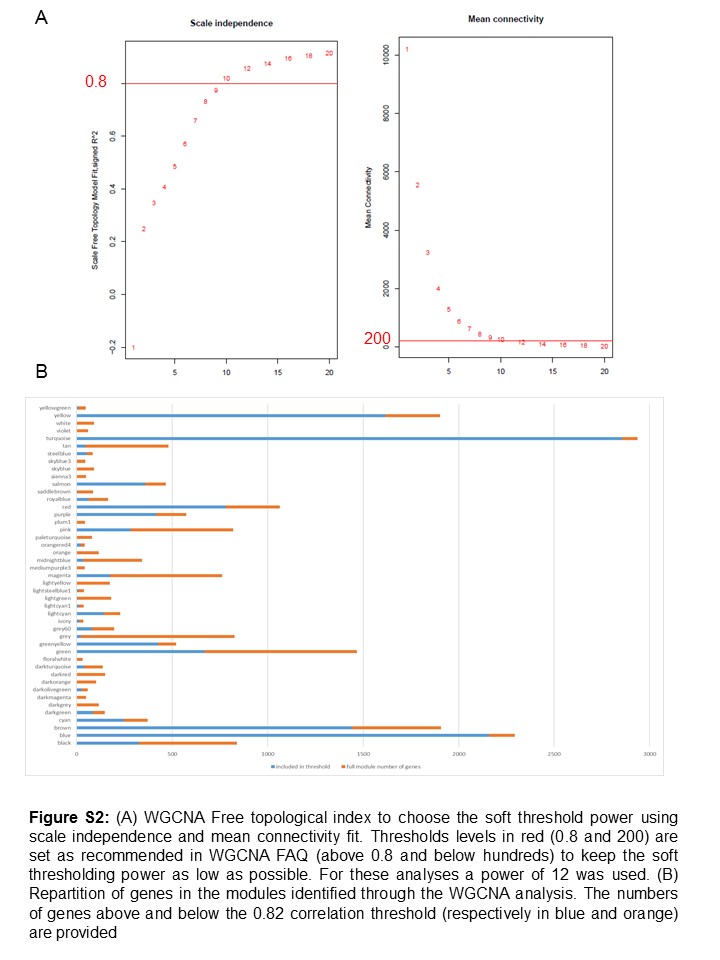

Supplement: Supplementary file 2 [file Image_2.JPEG]

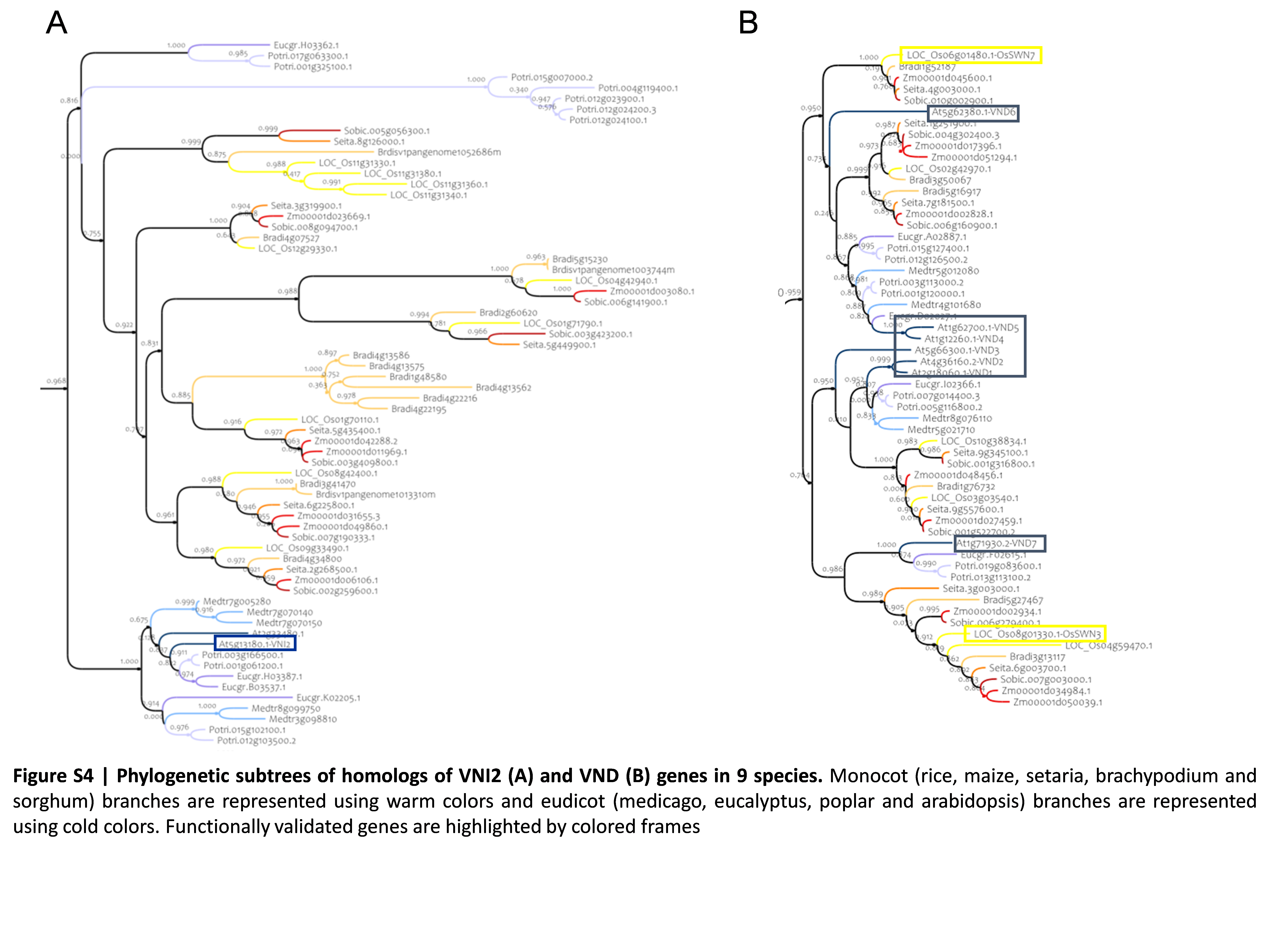

Supplement: Supplementary file 4 [file Image_4.TIF]
